# Exercise MR of Skeletal Muscles, the Heart, and the Brain

**DOI:** 10.1002/jmri.29445

**Published:** 2024-05-10

**Authors:** Melissa T. Hooijmans, Jeroen A.L. Jeneson, Harald T. Jørstad, Adrianus J. Bakermans

**Affiliations:** ^1^ Department of Radiology and Nuclear Medicine, Amsterdam University Medical Centers University of Amsterdam Amsterdam The Netherlands; ^2^ Department of Human Movement Sciences, Faculty of Behavioral and Movement Sciences Vrije Universiteit Amsterdam Amsterdam The Netherlands; ^3^ Center for Child Development and Exercise, Wilhelmina Children's Hospital/Division of Child Health University Medical Center Utrecht Utrecht The Netherlands; ^4^ Department of Cardiology Amsterdam University Medical Centers, University of Amsterdam Amsterdam The Netherlands

**Keywords:** ergometry, exercise intolerance, heart failure, human physiology, mitochondrial dysfunction, quantitative magnetic resonance imaging

## Abstract

Magnetic resonance (MR) imaging (MRI) is routinely used to evaluate organ morphology and pathology in the human body at rest or in combination with pharmacological stress as an exercise surrogate. With MR during actual physical exercise, we can assess functional characteristics of tissues and organs under real‐life stress conditions. This is particularly relevant in patients with limited exercise capacity or exercise intolerance, and where complaints typically present only during physical activity, such as in neuromuscular disorders, inherited metabolic diseases, and heart failure. This review describes practical and physiological aspects of exercise MR of skeletal muscles, the heart, and the brain. The acute effects of physical exercise on these organs are addressed in the light of various dynamic quantitative MR readouts, including phosphorus‐31 MR spectroscopy (^31^P‐MRS) of tissue energy metabolism, phase‐contrast MRI of blood flow and muscle contraction, real‐time cine MRI of cardiac performance, and arterial spin labeling MRI of muscle and brain perfusion. Exercise MR will help advancing our understanding of underlying mechanisms that contribute to exercise intolerance, which often proceed structural and anatomical changes in disease. Its potential to detect disease‐driven alterations in organ function, perfusion, and metabolism under physiological stress renders exercise MR stress testing a powerful noninvasive imaging modality to aid in disease diagnosis and risk stratification. Although not yet integrated in most clinical workflows, and while some applications still require thorough validation, exercise MR has established itself as a comprehensive and versatile modality for characterizing physiology in health and disease in a noninvasive and quantitative way.

**Evidence Level:**

5

**Technical Efficacy:**

Stage 1

Exercise is arguably the most common form of physical stress that humans encounter in daily life. One's ability to perform exercise or even daily‐life tasks is strongly associated with morbidity and all‐cause mortality, and is an important indicator if not predictor of physical health.^1^ Engaging in physical exercise is a voluntary action initiated by the brain, and set in motion through the recruitment of motor units that each consist of a motor neuron and its innervated skeletal muscle fibers. Such myofiber contraction triggers a cascade of biochemical and physiological responses, including the rise of cellular metabolism that leads to recruitment of the local microvasculature, and the cardiac and respiratory reserve, in order to sustain the desired mechanical task while maintaining energy homeostasis and pH balance in active muscle fibers. To do so, the musculoskeletal, cardiovascular, respiratory, and nervous systems must all work together seamlessly (Fig. 1).

**FIGURE 1:**
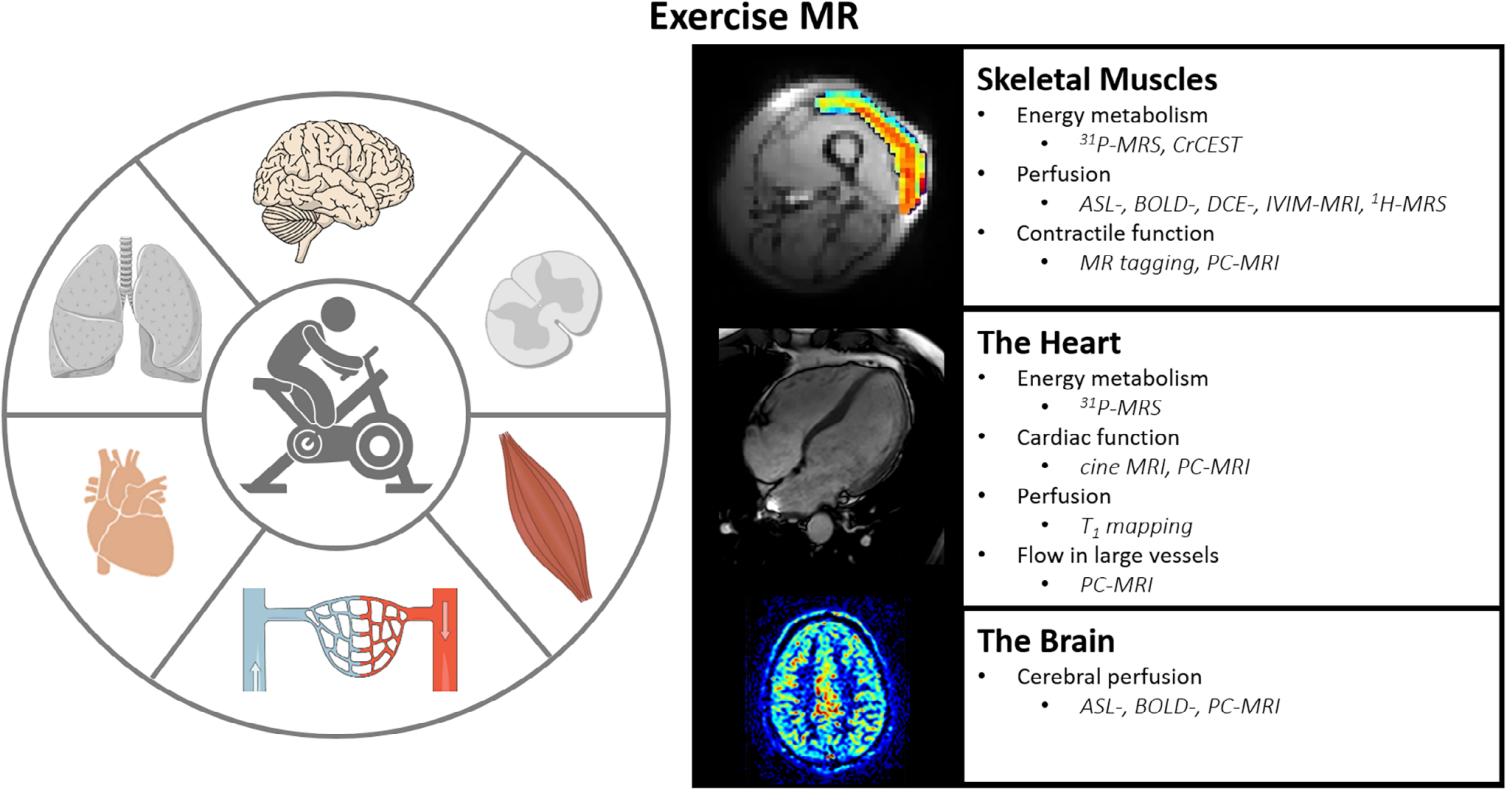
Schematic overview highlighting the involvement of skeletal muscles, the heart, and the brain to perform physical exercise as a complex interplay of the musculoskeletal, cardiovascular, respiratory, and nervous systems. Many aspects of human in vivo organ function, perfusion, and metabolism can be evaluated noninvasively with magnetic resonance (MR) techniques during exercise, eg, tissue oxygenation with blood oxygenation level‐dependent (BOLD) MRI of skeletal muscle, myocardial function with cine MRI, and cerebral perfusion with arterial spin labeling (ASL) MRI. Other readouts include proton MR spectroscopy (^1^H‐MRS), phosphorus‐31 MRS (^31^P‐MRS), chemical exchange saturation transfer effect of free creatine (CrCEST), dynamic contrast enhanced (DCE) MRI, intravoxel incoherent motion (IVIM) MRI, and phase‐contrast (PC) MRI. To date, essentially no or very limited work on exercise MR of the lungs or any other organs has been reported. Figure created with BioRender.com, and Servier Medical Art (https://smart.servier.com) under a Creative Commons Attribution 3.0 Unported license (https://creativecommons.org/licenses/by/3.0/).

In numerous pathophysiologic conditions, exercise cannot be sustained due to premature mechanical failure of contracting myofibers. Indeed, exercise intolerance is a hallmark of metabolic^2^ and neuromuscular disorders,^3^ as well as complex systemic diseases such as heart failure,^4^ post‐viral fatigue,^5^ and diabetes.^6^ Physical complaints typically present only during exercise, particularly in early stages of disease, and exercise intolerance may worsen as disease progresses. Currently, cardiopulmonary exercise testing (CPET) constitutes a central part of clinical workflows for evaluating the differential contributions to exercise intolerance of the peripheral, cardiovascular, and respiratory systems.^7^, ^8^ Exercise testing can also be used to elicit a pathophysiological response to help classify disease severity, eg, in heart failure with preserved ejection fraction (HFpEF)^9^ and peripheral artery disease.^10^ Such examinations aim to replicate the physical complaints experienced by the patient in daily life, and allow a direct evaluation of the underlying pathophysiology. The merit of exercise testing, beyond organ's anatomy, tissue perfusion and resting metabolic state, has thus been long recognized and accepted in the clinical practice. With an aging population and high prevalence of obesity and diabetes, the number of patients presenting with complaints related to reduced exercise capacity and exercise intolerance will increase. Moreover, there is mounting evidence that regular physical exercise improves musculoskeletal,^11^ cardiovascular,^12^ and cognitive health.^13^ We foresee growing demand for methods that can quantify the response of the human body to exercise stress will grow. Specifically, the combination of physiological stress with simultaneous readouts of in vivo organ function, perfusion, and metabolism that is possible with exercise magnetic resonance (MR) shows great promise to contribute to advancing our understanding of exercise physiology in health and disease.

Medical diagnostic imaging modalities typically require patient immobility during image acquisition, and MR imaging (MRI) is no exception. Given the relatively low sensitivity of MR and the consequent need for repeated acquisitions to collect sufficient signal for image reconstruction, much thought and effort has been put into mitigating motion artifacts that would otherwise reduce image quality. Yet, already in the early days of MR for in vivo human applications, its noninvasive nature and therewith its possibilities to acquire quantitative data in situ without disruptive biopsy procedures were utilized to study muscle energy metabolism during exercise in human extremities with phosphorus‐31 MR spectroscopy (^31^P‐MRS).^14^, ^15^ Driven by developments in hardware, acquisition strategies, and reconstruction algorithms, the use of both MRS and MRI for investigating exercise physiology has since evolved beyond those early ^31^P‐MRS studies of exercising human skeletal muscle in the 1980s.

In this review, we outline and discuss practical and physiological aspects of exercise MR. We present examples of how MR examination with exercise stress can shed light on human physiology, and how it can help to unmask otherwise undetectable pathophysiology. The acute impact of physical exercise on active skeletal muscles, the heart, and the brain is addressed, as well as the physiological and metabolic readouts that can be gained from studying these organs during recovery immediately after exercise. Finally, we reflect on how exercise MR stress testing can be a versatile platform for comprehensive, multi‐level, and multi‐organ investigations of both physiology and pathophysiology.

## Modes for Exercise MR Stress Testing

The majority of MR studies on acute effects of exercise have been conducted with physical exercise performed in *horizontal* body position, with the subject supine or prone on the MR table. Notable exceptions are some studies were subjects exercised in an upright position on a treadmill directly adjacent to the MR system, with measurements being done after quickly transferring the subject onto the table and into the MR scanner after exercise.^16^, ^17^ Other studies have utilized a *vertical* open‐bore 0.5 Tesla MR system for upright bicycling exercise.^18^, ^19^ With contemporary clinical MR systems typically configured with a horizontal bore, much effort has been put into the design of ergometers that are compatible with the strong magnetic field while still enabling physiological modes of prone or supine exercise within the confined space of the MR bore.^20^, ^21^, ^22^ The most simple but somewhat atypical mode of exercise is isometric handgrip exercise. It avoids body movement, but induces only a small increase in heart rate at low‐intensity exercise. Many investigations focus on isometric or concentric muscle contraction at a predefined percentage of maximum voluntary contractile force, either until exhaustion or for a predetermined duration. While such single‐limb or single‐muscle exercise elicits a *local* physiological response, these modes fall short in realistically capturing any daily‐life physical activity. Moreover, such exercises only involve a small muscle mass that is insufficient to trigger a physiologically realistic systemic cardiovascular response,^23^ which requires recruitment of a much larger muscle mass.^24^ In‐magnet leg‐bicycling^21^, ^25^ or arm‐bicycling^26^, ^27^ paradigms have been developed for use in clinical MR systems. These modes provide more realistic surrogates for whole‐body exercise that can be combined with dynamic MR acquisitions to study systemic aspects of human exercise physiology. Still, the horizontal body posture will impact the hemodynamic response^28^ (eg, due to differences in gravity‐induced hydrostatic pressures that affect cardiac preload), skeletal muscle recruitment and perfusion, and exercise capacity.^29^ These effects should be considered when comparing MR‐measured parameters against more clinically accepted exercise stress tests such as CPET that are conducted in an upright position.

## Physical Exercise and Skeletal Muscles

Exercise MR stress testing has been pivotal for over 50 years in offering quantitative in vivo data for the study of skeletal muscle energy metabolism, peripheral microvascular function, and muscle contraction dynamics.^30^ Despite its demonstrated merit in many clinical applications, exercise MR stress testing of skeletal muscles has not (yet) been implemented into clinical routines. For various reasons, many studies have predominantly examined muscles in the lower leg, i.e., the medial and lateral gastrocnemius and tibialis muscles, with single‐leg (rhythmic) dorsi‐/plantarflexion motor tasks.^31^ Other single‐limb exercise modes include knee flexion or extension exercise that recruits upper leg muscles, or handgrip exercises that involve flexor muscles of the lower arm.^32^ Such exercise stress tests are typically performed in a rest‐exercise‐recovery paradigm, comprising a baseline rest period, followed by an exercise period and subsequent recovery. The exercise phase can adopt either an intermittent paradigm, consisting of a single muscle contraction followed by an interval during which MR data are recorded, or a continuous exercise paradigm, where no intervals of exercise cessation are incorporated for data acquisition. The latter paradigm poses a substantial challenge in obtaining meaningful quantitative MR data *during* exercise, even if acquisitions are triggered to exercise rhythm.

Standardized exercise MR stress testing protocols to investigate specific physiological effects in skeletal muscle are essentially lacking, while literature reports frequently provide limited details regarding type of exercise, intensity, and duration. Furthermore, despite the growing availability of dedicated MR‐compatible ergometers designed specifically for evaluating skeletal muscle,^33^, ^34^ many studies still rely on unique, in‐house built MR‐compatible ergometers.^32^ Such variations between laboratories and between studies need to be considered when interpreting and comparing reports on exercise MR stress testing of skeletal muscle.

### Skeletal Muscle Energy Homeostasis and pH Balance During Exercise

**FIGURE 2:**
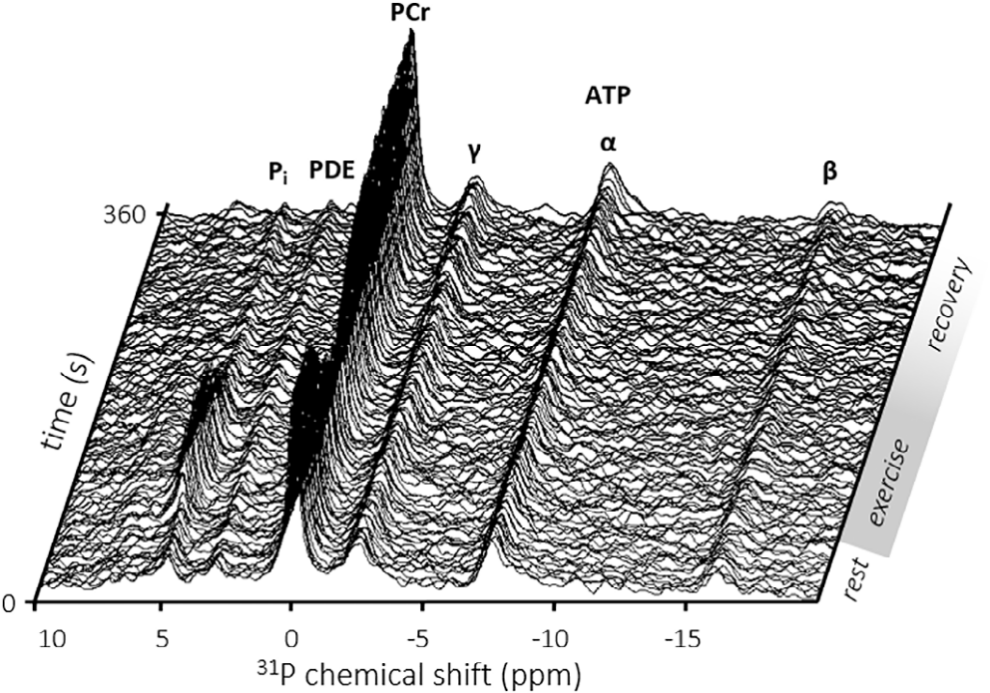
Time‐resolved series of ^31^P‐MR spectra of the calf musculature of a healthy subject (female; 67 years) acquired at rest, during plantarflexion exercise, and during subsequent recovery at a temporal resolution of 3 s. Note the exercise‐induced 41.5% depletion of phosphocreatine (PCr) and concomitant rise of inorganic phosphate (P_i_) that recovered (PCr recovery time constant, 25.4 s) upon cessation of exercise. α, β, γ‐ATP = α‐, β‐, γ‐phosphate groups in adenosine 5′‐triphosphate; PDE = phosphodiesters. Reproduced from Bakermans et al^197^ with permission from John Wiley & Sons, Inc. under a Creative Commons BY‐NC license (https://creativecommons.org/licenses/by‐nc/4.0/).

Adenosine 5′‐triphosphate (ATP) metabolism in in vivo skeletal muscle can uniquely be studied noninvasively during exercise with time‐resolved ^31^P‐MRS (Fig. 2). Dynamic ^31^P‐MRS allows the direct detection of inorganic phosphate (P_i_) and phosphocreatine (PCr) concentration kinetics, as well as estimation of intracellular tissue pH.^31^ Muscle contraction causes accumulation of P_i_ and depletes the cytosolic PCr pool, while mitochondrial oxidative metabolism replenishes this ATP energy buffer by producing ATP and via the creatine kinase shuttle in the presence of vascular oxygen supply. As such, the PCr recovery rate after aerobic exercise serves as a surrogate measure of in vivo muscle oxidative capacity,^35^ comprising intrinsic mitochondrial function, number of mitochondria, and oxygen transport. A comprehensive overview of relevant ^31^P‐MRS‐derived outcome parameters and of how to quantify those in skeletal muscle is provided elsewhere.^31^

Many early studies employed time‐resolved *pulse‐acquire surface coil‐localized* ^31^P‐MRS to characterize mitochondrial ATP synthesis in health and disease. For instance, early investigations of patients with peripheral artery disease reported greater PCr depletion and intracellular acidosis during exercise, followed by delayed metabolic recovery relative to healthy subjects. Interestingly, limb blood flow measured by venous occlusion plethysmography appeared to be normal in those patients.^36^, ^37^ A similar approach was combined with incremental concentric plantarflexion exercise that induced a 50% PCr depletion in patients with dermatomyositis (*n* = 9) and polymyositis (*n* = 5) to detect substantially longer PCr and adenosine diphosphate (ADP) recovery half‐times compared with healthy volunteers,^38^ which can be indicative of mitochondrial dysfunction. Proton efflux, derived from ^31^P‐MRS‐based pH estimates, however, was reduced in those patients, indicating that mitochondrial dysfunction was likely secondary to impaired blood supply to the musculature. Those and other^39^ studies illustrate how dynamic ^31^P‐MRS during both exercise and subsequent recovery provides distinct quantitative readouts on in vivo muscle oxidative capacity in disease.

**FIGURE 3:**
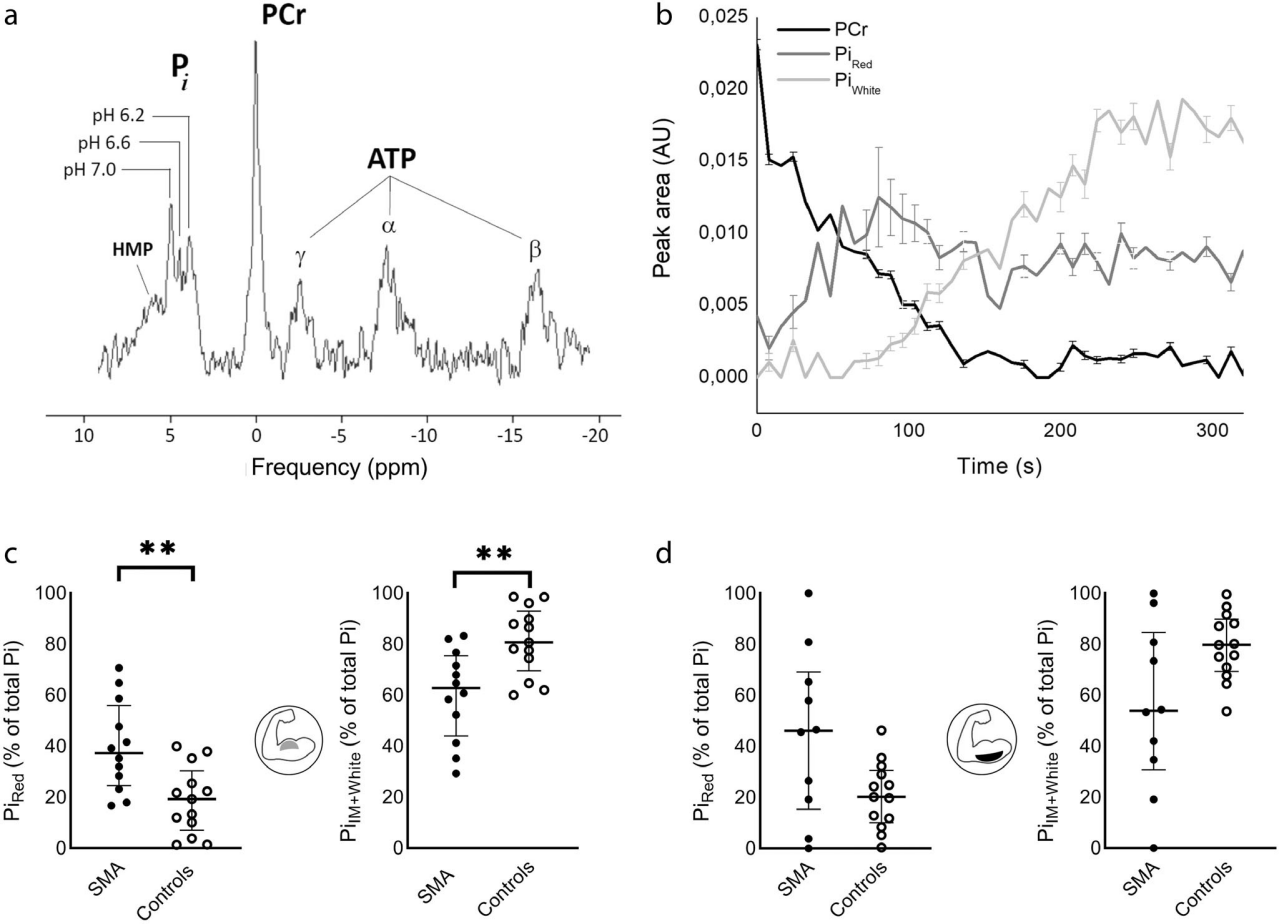
Phosphorus‐31 magnetic resonance spectroscopy (^31^P‐MRS) of the proximal arm musculature during supine arm‐bicycling exercise. (**a**) ^31^P‐MR spectrum of the biceps brachii of a healthy subject acquired 1 minute after onset of supine arm‐bicycling exercise, revealing splitting of the inorganic phosphate (P_i_) resonance peak that reflects myofibers operating under different pH values. (**b**) Phosphocreatine (PCr) and P_i_ concentration kinetics in red and white myofibers of the biceps brachii of a patient with spinal muscular atrophy (SMA) during arm‐bicycling until exhaustion. (**c**) Fractional signal amplitudes of P_i_ in red myofibers, and in intermediate + white myofibers as a percentage of total P_i_ in the biceps brachii and triceps brachii (**d**) muscles of the individual SMA patients and healthy subjects at end‐exercise. Note the substantially larger fraction of red myofibers in the patients with SMA. α, β, γ‐ATP = α‐, β‐, γ‐phosphate groups in adenosine 5′‐triphosphate; AU = arbitrary units; HMP = hexose monophosphate. **P < 0.01. Reproduced from Habets et al^27^ with permission from Oxford University Press under a Creative Commons BY‐NC license (https://creativecommons.org/licenses/by‐nc/4.0/).

Various studies with time‐resolved ^31^P‐MRS of muscle energy homeostasis and pH balance observed high‐ and low‐pH milieus within skeletal muscles of healthy subjects during high‐intensity exercise, manifesting as an exercise‐induced and pH‐mediated “splitting” of the P_i_ resonance peak.^40^, ^41^ It was suggested that such pH heterogeneity within active muscles reflects metabolic differences between oxidative and glycolytic muscle fibers. This particular explanation for pH heterogeneity has often been ambiguous, because signal localization to a single working muscle could often not be ascertained.^31^ Yet, elegant experiments by Mizuno et al that used curare to selectively block motor units showed that P_i_ peak splitting may indeed inform on fiber type recruitment, provided that ^31^P‐MR signal can be obtained from a single working muscle with adequate blood flow.^42^ This phenomenon proved valuable in a study of upper arm muscles of patients (*n* = 15) with spinal muscular atrophy (SMA). During arm‐bicycling exercise inside a 3 Tesla MR system, a differential intramuscular P_i_ accumulation in red (i.e., slow‐twitch oxidative) vs. white (i.e., fast‐twitch glycolytic) and intermediate myofibers was observed (Fig. 3), identifying white‐to‐red fiber type remodeling of the residual upper arm musculature in those patients.^27^ As such, those data demonstrated how noninvasive ^31^P‐MRS can provide evidence of the selective degeneration of fast and strongly contracting myofibers in patients with SMA, ultimately leading to muscle atrophy and weakness.

While pulse‐acquire surface coil localization remains a widely favored method owing to its accessibility and effectiveness, it is important to acknowledge that signal obtained with this approach reflects all tissue within the sensitive area of the surface coil. Therefore, precise coil positioning is crucial for obtaining accurate and muscle‐specific data. Given the non‐uniform spatial distribution of both muscle physiology (eg, myofiber types) and pathology,^43^ resolving spatial information with ^31^P‐MRS has become increasingly important. A variety of approaches emerged to improve spatial resolution, including two‐dimensional (2D) chemical shift imaging,^44^, ^45^ multi‐coil setups to measure along the length of individual muscles,^46^ multi‐voxel localized MRS acquisitions^47^ to assess multiple muscles simultaneously,^48^ and ^31^P‐MRI to map individual metabolites.^49^ A challenge of such spatially resolved experiments is to achieve sufficient temporal resolution for following metabolite (typically P_i_ and PCr) concentration kinetics during exercise and recovery. Using multiple frequency‐selective radiofrequency (RF) excitations for ^31^P‐MRI at 7 Tesla, PCr and P_i_ signals were sampled in an interleaved fashion during isometric plantarflexion exercise at a spatial resolution of 2 mL and a temporal resolution of 6 s.^50^ Another study used multi‐voxel ^31^P‐MRS to evaluate how muscle oxidative capacity changes with age, revealing substantially slower P_i_ and PCr recovery kinetics in the calf muscles of elderly subjects (64.6 ± 5.8 years) after isometric plantarflexion exercise.^51^ In patients with heart failure, a combined ^31^P‐MRS and ^31^P‐MRI approach at 3 Tesla was used to reveal reduced PCr recovery rates in soleus and gastrocnemius muscles after dynamic plantarflexion exercise compared with healthy subjects.^52^ Those studies highlight how localized readouts can assess differences in oxidative capacity and intracellular tissue pH among individual muscles, emphasizing the added value of acquisitions at higher spatial resolution beyond pulse‐acquire surface coil‐localized ^31^P‐MRS. Moreover, it is practically impossible to replicate the performed exercise intensity for obtaining data on multiple muscle separately in repeated sessions.

**FIGURE 4:**
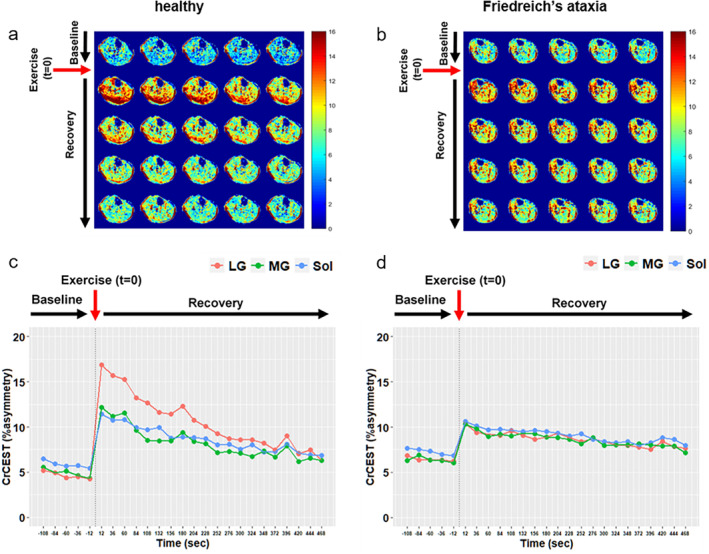
Series of maps of the chemical exchange saturation transfer effect of free creatine (CrCEST) acquired from the lower leg of a healthy (**a**) subject (male; 25 years) and a patient (male; 30 years) with Friedreich's ataxia (**b**) at rest and during recovery after plantarflexion exercise. Maps are ordered chronologically from left to right, then top to bottom, respectively. The color bars indicate the CrCEST signal intensity proportional to the tissue free creatine concentration. Note the free creatine concentration kinetics (**c**, **d**) for several different muscles, revealing prolonged recovery times in Friedreich's ataxia. LG = lateral gastrocnemius; MG = medial gastrocnemius; Sol = soleus. Reproduced with adapted labels from original by Schur et al^58^ under a Creative Commons BY license (https://creativecommons.org/licenses/by/4.0/).

Alternative MR methods for noninvasively evaluating skeletal muscle energy metabolism are ^31^P‐MRS magnetization transfer (MT)^53^ and measuring the chemical exchange saturation transfer effect of free creatine (CrCEST) with ^1^H‐MRI.^54^ MT experiments have been used primarily to estimate the exchange flux between P_i_ and ATP as a measure of mitochondrial function in resting muscle, which may be difficult to interpret.^55^, ^56^ Instead, CrCEST has been used to measure recovery kinetics of free creatine, a byproduct of immediate ATP regeneration through PCr buffering, after exercise. In patients (*n* = 13) with genetic mitochondrial disorders, CrCEST at 7 Tesla revealed prolonged recovery times of free creatine returning to baseline levels in the medial gastrocnemius muscle after in‐magnet plantarflexion exercise, which is indicative of impaired oxidative capacity in these patients.^57^ At 3 Tesla, much slower free creatine recovery kinetics were found in the lateral gastrocnemius (exponential decline time constant, 274 s vs. 138 s, *P* = 0.01) in adults (*n* = 11) with Friedreich's ataxia compared with healthy subjects (*n* = 25),^58^ similarly suggesting reduced oxidative capacity (Fig. 4). The primary advantages of employing CrCEST instead of ^31^P‐MRS are the high signal‐to‐noise ratio that comes with ^1^H‐MRI, superior spatial resolution, and no requirement for dedicated ^31^P‐MR hardware. Conversely, CrCEST solely probes free creatine, is susceptible to motion artifacts, and is constrained by a limited temporal resolution of 20–30 s.

### Skeletal Muscle Perfusion

In order to sustain the mechanical function of skeletal muscle, adequate regulation of muscle perfusion to meet the local demand of oxygen and nutrients is critical. Many diseases are characterized by impairments of local peripheral microvascular function, which is often not detectable in resting skeletal muscle due to low perfusion of inactive muscles. In active muscles, the demand for oxygen and substrates increases, leading to an overall increase in muscle perfusion by 500%–1000%.^59^ As such, assessments of perfusion dynamics during and after exercise can serve as a surrogate measure for microvascular function, reflecting key aspects of the convective oxygen transport chain.

Various MRI techniques probe different components of this process, such as blood oxygenation level‐dependent (BOLD) MRI, arterial spin labeling (ASL) MRI, and dynamic contrast enhanced (DCE) MRI that requires the infusion of a gadolinium‐based contrast agent. Whereas ASL‐ and DCE‐MRI probe tissue perfusion directly and quantitatively, BOLD‐MRI is a composite readout that is sensitive to a variety of physiological processes including blood flow, blood volume, and level of oxygenation. Notably, in BOLD‐MRI dynamic changes in signal intensity or T_2_* relaxation time constants are typically expressed relative to a baseline condition rather than in physiological units. Several studies have explored the origin of BOLD contrast in healthy muscle tissue by comparing BOLD‐MRI with other modalities such as near‐infrared spectroscopy (NIRS),^60^ transcutaneous oxygen pressure,^61^ and skin laser Doppler flowmetry.^62^ Those investigations demonstrated good measurement agreement between modalities in terms of hyperemic response magnitude and timing. Notably, a proposed model by Elder et al^63^ revealed that BOLD contrast predominantly reflects intravascular rather than extravascular effects.^64^ Although ASL‐MRI can quantify skeletal muscle perfusion in response to vasoactive stimuli, measuring perfusion in resting skeletal muscle is challenging due to the low signal‐to‐noise ratio resulting from minimal blood flow at rest. Additionally, generating a quantitative perfusion map with ASL‐MRI requires the subtraction of control‐ and label‐images, which is prone to exercise‐induced displacement artifacts. Lastly, the conventional implementation of ASL‐MRI relies on a single post‐labeling delay, thus presuming that arterial blood travels at a uniform speed, which may not accurately reflect actual physiology during exercise.

By deriving quantitative measures from dynamic series of BOLD‐, ASL‐ and DCE‐MRI, valuable insights on microvascular function in healthy and diseased skeletal muscle have been obtained during and after exercise. Using ASL‐MRI at 3 Tesla, a blunted hyperemic response evidenced by a lower % change, a lower gradient and a longer time‐to‐peak after isometric plantarflexion contractions was found in patients with peripheral artery disease,^65^ indicating impaired microvascular function in the calf musculature. Other work investigated microvascular function with BOLD‐MRI at 3 Tesla during and after submaximal (50% of maximal voluntary contraction) and maximal isometric dorsiflexion exercise in obese subjects (*n* = 8) and in patients (*n* = 8) with type 2 diabetes mellitus. Compared with lean age‐matched subjects (*n* = 8), blood volume was lower after maximal but not submaximal contractions in the extensor digitorum longus muscle of subjects with obesity and diabetes, suggesting exercise intensity‐dependent structural or functional impairment of the microvasculature.^66^

**FIGURE 5:**
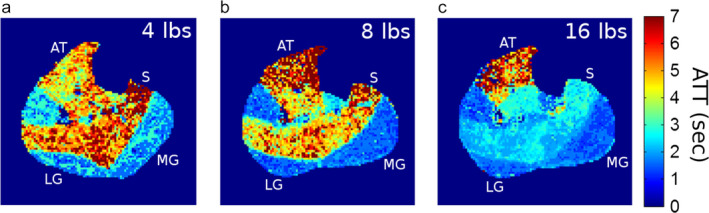
Maps of the arterial transit time (ATT) derived from dynamic contrast enhanced (DCE) MRI in calf muscles of a young healthy subject after dynamic plantarflexion exercise against three different loads: 4 lbs (**a**), 8 lbs (**b**), and 16 lbs (**c**). Note how ATT values vary substantially between the anterior and posterior compartments, the individual triceps surae muscles (gastrocnemii and soleus), and with different workloads, showing lower ATT values (i.e., faster perfusion) with more active muscles. AT = tibialis anterior; LG = lateral gastrocnemius; MG = medial gastrocnemius; S = soleus. Reproduced from Conlin et al^67^ under a Creative Commons BY license (https://creativecommons.org/licenses/by/4.0/).

The arterial transit time (ATT) quantifies the delivery of blood to the muscles by measuring the time delay for arterial blood to travel into the tissue microvasculature. Using DCE‐MRI, progressive shortening of the ATT was found in healthy volunteers during recovery after plantarflexion exercise against increasing workloads (Fig. 5).^67^ Those findings challenge the conventional implementation of ASL‐MRI^68^ and emphasize the importance of adequate or multiple post‐labeling delays. In healthy volunteers, ASL‐MRI with multiple post‐labeling delays was used to characterize recovery effects after dynamic dorsiflexion exercise, revealing proximally elevated perfusion and a shorter ATT compared with distal tissue.^69^ Those data emphasize that multiple post‐labeling delays are needed to quantify skeletal muscle perfusion with ASL‐MRI, and highlight how microvascular function heterogeneity within individual muscles can be studied with exercise MR stress testing of skeletal muscles. Such observations are particularly important for clinical applications, given that pathology can considerably affect ATT.^67^

**FIGURE 6:**
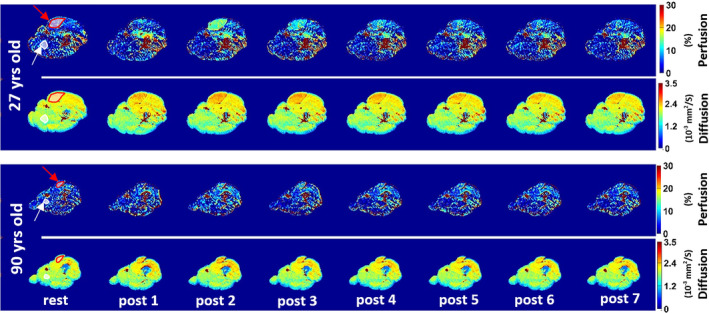
Intravoxel incoherent motion (IVIM) MRI‐based perfusion fraction and water diffusion maps from the upper leg of a young (27 years) and an old (90 year) subject at rest and after dynamic knee extension exercise at 3.25‐minute intervals. Regions of interest for the active rectus femoris muscle (red) and for a part of the inactive adductor magnus muscle (white) are indicated. Note the pronounced difference in perfusion fraction between the young and old subjects after exercise. Reproduced from Adelnia et al^71^ with permission from John Wiley & Sons, Inc.

Alternatively, intravoxel incoherent motion (IVIM) MRI utilizes spin dephasing‐induced signal loss to visualize and quantify various perfusion‐related parameters including perfusion fraction (*f*_*p*_), water diffusion (*D*) and pseudo‐diffusion (D*).^70^ Compared with BOLD‐, ASL‐ and DCE‐MRI, IVIM‐MRI has a substantially lower temporal resolution of 2–3 minutes. Similar to ASL‐MRI, IVIM‐MRI measurements at rest are challenged by low resting‐state perfusion of skeletal muscle. Only a few studies used in‐magnet exercise, because contrast for IVIM‐MRI is typically generated by comparing baseline and post‐exercise conditions. Using IVIM‐MRI at 3 Tesla following in‐magnet incremental knee extension exercise, a blunted increase (+18 ± 16%) in perfusion was observed (Fig. 6) in upper leg muscles of elderly (60–90 years; *n* = 4) compared with healthy young subjects (+37 ± 12%; 21–30 years; *n* = 4).^71^ Those results are in line with other reports that revealed aging‐related reduced muscle perfusion as measured with ASL‐MRI after moderate‐intensity plantarflexion exercise in older subjects,^72^ and a blunted hyperemic response after vascular occlusion as measured with BOLD‐MRI.^73^

Beyond assessments of microvascular perfusion, (de)oxygenation of myocytes can be probed through the detection of deoxymyoglobin (DMb) with ^1^H‐MRS.^74^ At rest, typically no DMb signal can be observed because myoglobin will be fully oxygenated. Instead, during arterial occlusion^74^ or after maximal exercise,^75^ a clear DMb signal arises. Such DMb signal is a composite measure capturing aspects of both convective and diffusive oxygen transport chains, and holds potential as a quantitative parameter that reflects tissue reoxygenation (deficits).^76^ Indeed, a study in patients with glycogen storage disease used an interleaved ^1^H‐MRS, ASL‐ and BOLD‐MRI approach at 4 Tesla to reveal delayed reoxygenation and concomitantly blunted microvascular response after dynamic plantarflexion exercise, suggesting that reduced tissue perfusion may contribute to impaired oxidative capacity and exercise intolerance in those patients.^77^ Compared with other methods outlined above, the detection of DMb with ^1^H‐MRS suffers from low sensitivity and does not provide spatial information. As such, it may not be the readout of choice during an exercise MR stress test. Indeed, tissue oxygenation states may now preferably be assessed clinically and much more reliably using novel optical equipment, i.e., with diffuse correlation spectroscopy^78^, ^79^ and photo‐acoustic imaging.^80^ However, multi‐parametric dynamic MR that includes the acquisition of signal from DMb has recently been implemented on a clinical setup, allowing the evaluation of tissue (re)oxygenation in conjunction with, rather than at the expense of, other readouts such as BOLD‐MRI and ^31^P‐MRS.^81^

As an alternative to physical exercise, cuff occlusion can be used for ischemia‐hyperemia paradigms in order to noninvasively evaluate microvascular reactivity with MRI. Compared to exercise protocols, cuff occlusions are highly reproducible, well‐controlled, and do not rely on active participation from the subject. Although such occlusions do not trigger the hemodynamic and metabolic responses similar to daily‐life physical activities, it may be a valuable alternative for obtaining data on microvascular function in patients who are unable to perform exercise inside an MR scanner. Currently, no standardized cuff occlusion protocols exist, hampering fair comparisons of data from different laboratories and different studies.

Several studies have assessed the precision of these MRI techniques for measuring muscle perfusion,^81^, ^82^, ^83^ yielding variable results for both ischemia‐hyperemia and exercise protocols. To advance the clinical utility of these techniques, a clear perspective of the measurement precision and reproducibility is essential. Precision can be improved by enhancing signal‐to‐noise ratios, and temporal and spatial resolution, potentially through machine learning and accelerated imaging approaches, while reproducibility will improve with protocol standardization. More details about how techniques for functional MRI of skeletal muscles relate to one another and to muscle physiology, and more examples of clinical applications can be found elsewhere.^83^, ^84^

### Skeletal Muscle Contractile Function

Conventionally, muscle contractile function has been evaluated by surface or needle electromyography‐based measurements, assessing a muscle's *electrical* response to incoming stimuli from motor‐neurons resulting in the activation of single or multiple motor‐units.^85^ With MRI, contractility measures in terms of tissue *displacements* and derived strains, velocities and strain rates resulting from these electrical activities in active skeletal muscle are visualized and quantified. This is particularly relevant for investigating degenerative neuromuscular disorders, which are characterized by a lower quality of the remaining muscle tissue that is likely caused by altered contractile function.

The spatial resolution of MRI is typically in the order of millimeters, and precluding discerning individual sarcomeres, but it allows the characterization of muscle fascicle displacements and whole‐muscle mechanics. To date, only one study compared surface electromyography with MRI during isometric plantarflexion exercise, reporting good agreement (0.84 < *r* > 0.88) between contraction dynamics measured by 2D velocity‐encoded phase‐contrast MRI and surface electromyography data.^86^

**FIGURE 7:**
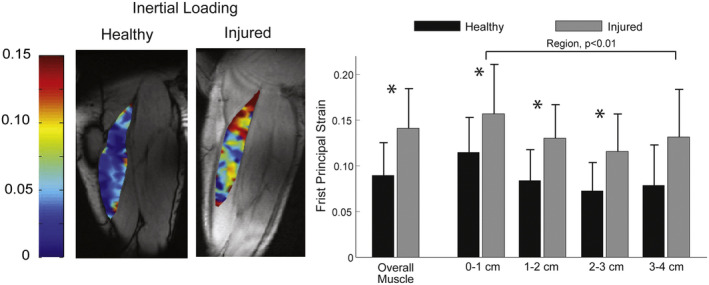
First principal strain maps from the biceps femoris during peak knee extension under inertial loading conditions overlaid on coronal anatomical MR images obtained in the upper leg of an uninjured athlete and an athlete with prior hamstring injury. Larger strains were observed in the tissue nearest to the myotendinous junction. Note that strains were substantially larger in athletes with prior hamstring injuries compared to uninjured subjects. *P < 0.05. Reproduced from Silder et al^91^ with permission from Elsevier.

By measuring displacements and velocities using MRI in skeletal muscle during exercise, quantitative estimates of muscle contraction dynamics can be obtained.^87^ From such velocity and displacement data, strain rate quantifies the change in deformation over time, while principal strains are derived by integrating strain rate over time. The latter calculation is valid under the assumption that the voxels of the acquired slice do not move out of the imaging plane. This can often not be ascertained for concentric and eccentric contractions, and therefore most of the displacement‐ and velocity‐encoded MR measurements are typically conducted during isometric contractions that vary from 5% to 60% of maximal voluntary contraction. The sensitivity of such velocity‐ and displacement‐encoded MR techniques in relation to muscle contraction intensity was demonstrated in healthy subjects, detecting contractile heterogeneity between and within individual muscles.^88^, ^89^, ^90^ Those techniques provided valuable insights on muscle contractile function in clinical conditions. Using 2D velocity‐encoded phase‐contrast MRI at 1.5 Tesla, it was shown (Fig. 7) that athletes with a prior hamstring injury exhibited higher principal strains in the whole muscle as well as near the proximal myotendinous junction during active hamstring lengthening and shortening contractions.^91^ Those results suggest that post‐injury remodeling may adversely affect local tissue mechanics and could contribute to the high re‐injury risk in those athletes. With a similar approach at 3 Tesla, lower strain rates were measured during isometric plantarflexion exercise in elderly, and attributed to aging‐related remodeling of the extracellular matrix.^92^ In disuse atrophy induced by lower limb suspension in healthy volunteers, strain rates during isometric plantarflexion contractions decreased in the myofiber cross‐sectional direction,^93^ which was associated with loss of maximal contractile force. Those findings suggest extensive extracellular matrix remodeling in disuse atrophy that affects muscle contractility, illustrating how MRI can noninvasively inform on muscle mechanics.

**FIGURE 8:**
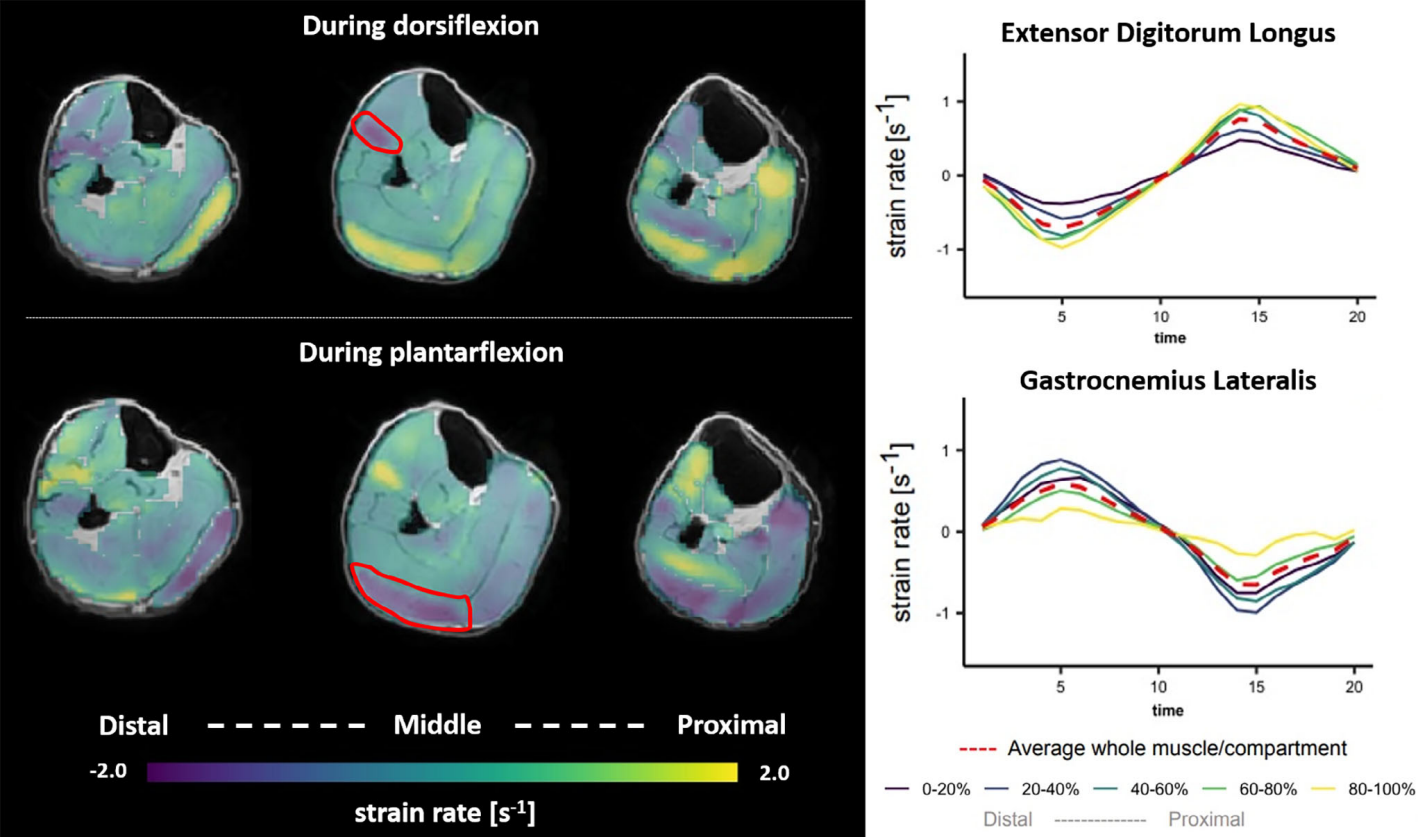
Cross‐sectional maps of strain rate along the myofibers during maximal dorsiflexion (top) and maximal plantarflexion (bottom) of a distal (segment 20%–40%), middle (segment 40%–60%), and proximal (segment 60%–80%) slice of the lower leg. Line graphs (right) showing average strain rates along the fiber in the extensor digitorum longus (top) and gastrocnemius lateralis (bottom) muscles throughout the unloaded exercise cycle for the five segments. Note the generally antagonistic behavior of the anterior and posterior compartments of the lower leg during exercise, as well as the difference between the individual segments. See Hooijmans et al^98^ for details.

Until now, studies predominantly used single or several imaging planes with 2D tensors to mitigate long acquisition times typically required for three‐dimensional (3D) volumetric acquisitions, even though it is known that both architectural and functional characteristics vary between as well as within individual muscles.^94^, ^95^ Furthermore, it is generally impossible to align the imaging plane precisely with all muscle fibers. Advancements in acquisition and reconstruction strategies enabled accelerated MRI for facilitating multi‐slice and volumetric whole‐muscle evaluations (Fig. 8) of the 3D strain rate tensor with appropriate temporal resolution (10 msec) and acceptable acquisition times.^96^, ^97^, ^98^ More research in healthy subjects and patients is essential to truly determine the added value of evaluating whole‐muscle volumes and contraction dynamics during exercise in 3D.

As an alternative to probing tissue displacements, the visualization and quantification of contraction‐induced changes in the skeletal muscle microstructure could serve as an outcome measure for contractile function. However, conventional diffusion tensor imaging (DTI) MRI acquisitions for mapping muscle microstructure prove unsuitable for this purpose, given their high sensitivity to bulk motion of both voluntary and involuntary contractions, which leads to substantial signal voids. Recently, Mazzoli et al used an oscillating gradient spin echo (OGSE) diffusion encoding strategy.^99^ By employing short diffusion weighting times and trapezoid‐cosine OGSE waveforms, this technique allowed the quantification of tissue water diffusion properties in actively contracting skeletal muscle (isometric contractions; acquisition time 90 s) of healthy volunteers. As such, this approach holds potential for noninvasively investigating contraction abnormalities in patients with neuromuscular disorders. Conversely, diffusion‐weighted MRI can also utilize the signal voids that are introduced by muscle contraction^100^ for “motor unit MRI”. This emerging area of research has recently been reviewed elsewhere.^101^

## Physical Exercise and the Heart

Exercise stress testing of the cardiovascular system, and the heart in particular, constitutes an important part of the clinical evaluation of patients with exercise‐related complaints. While conventional (i.e., with electrocardiography [ECG] only) exercise tests are no longer part of the standard work‐up for ischemic heart diseases, CPETs that measure respiratory oxygen uptake, carbon dioxide production, systemic blood pressures, and ECG are increasingly being used to evaluate symptomatic patients and to quantify cardiorespiratory fitness.^8^ Transthoracic echocardiography is the imaging workhorse of clinical cardiology to noninvasively evaluate and quantify cardiac performance. Under exercise stress, the image quality of transthoracic echocardiography may deteriorate substantially, particularly in obese subjects, and views of the right ventricle (RV) are difficult to obtain. Such evaluations with MRI under exercise stress are challenging as well. Yet, already in an early stage of the rise of MRI as a clinical imaging modality, devices were designed that would allow for exercise MR stress testing of the heart^20^ as a potential alternative to pharmacological stress. The strong magnetic field as well as the narrow bore of the MR system put substantial constraints on the design and construction of such devices, limiting the flexibility and increasing the costs of this approach. Likewise, noninvasive blood pressure measurements cannot be obtained with standard monitors. In addition, safety^102^ and technical^103^ concerns can limit the feasibility and efficacy of exercise MR stress testing in patients with implantable electronic devices such as pacemakers and defibrillators. Taken together, these constraints make MRI under exercise stress technologically demanding. Yet, four decades of technological advances have now brought the field to an era where MR‐compatible ergometers are commercially available,^104^, ^105^ supine bicycling exercise stress testing is included in guidelines on standardized cardiovascular MRI protocols,^106^ and (patho)physiological cardiovascular responses to exercise have been investigated with a broad range of noninvasive MR readouts.^107^

### Blood Flow in the Great Vessels

**FIGURE 9:**
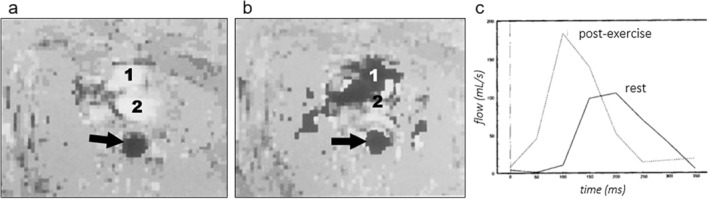
Peak systolic velocity maps of the descending thoracic aorta (arrow) and the right (1) and left (2) ventricular outflow tracts acquired at 0.5 Tesla at rest (**a**) and after exercise (**b**), showing blood flow velocity in cranial and caudal directions in light and dark shades of gray, respectively. Aliasing at high velocities caused the ventricular outflow tracts to appear black after exercise. Note the phasic nature of systolic blood flow in the aorta, and the elevated peak flow after exercise (**c**). Reproduced and relabeled from Mohiaddin et al^108^ with permission from John Wiley & Sons, Inc. © SMR, 1995.

The first MRI estimates of cardiovascular performance under exercise conditions were obtained from measurements of blood flow in the great vessels. Using a 0.5 Tesla MR system and an apparatus for supine in‐magnet pedaling exercise, velocity maps of the descending thoracic aorta were obtained in healthy volunteers (*n* = 10) at rest (heart rate, 68 ± 6 beats/min) and immediately after exercise (101 ± 12 beats/min).^108^ At a temporal resolution of 50 msec and an in‐plane spatial resolution of 5 × 5 mm^2^, the phasic nature of systolic aortic blood flow was demonstrated, revealing a >40% elevation of mean and peak flow immediately after exercise (Fig. 9).

At 1.5 Tesla and with a commercially available supine bicycle ergometer, 2D phase‐contrast MRI (1.5 × 1.5 mm^2^ in‐plane spatial resolution, 28 msec temporal resolution) of the abdominal aorta showed an increased flow from 1.4 ± 0.3 L/min at rest (65 ± 7 beats/min) to 7.9 ± 1.1 L/min after exercise (135 ± 22 beats/min) at 131 W.^109^ Retrograde flow present at rest disappeared at higher exercise intensity. Measurements were obtained during breath holds immediately after exercise, in order to minimize motion‐induced distortions of the ECG signal that was used for triggering, while accepting that heart rate drops rapidly^110^ by <14% after high‐intensity exercise in those young (27 ± 2 years; *n* = 9) healthy volunteers.^109^ This approach could potentially be used for assessments of the pulse wave velocity as a surrogate measure for vascular stiffness. Notably, descending aorta pulse wave velocity after exercise correlated positively with age (22–75 years; *n* = 50, *r* = 0.63, *P* < 0.001),^111^ and main pulmonary arterial wall stiffness increased in response to acute exercise^112^ in healthy volunteers. Those data suggest that evaluations of the great vessels under exercise stress can provide information on *vascular* function, eg, in atherosclerosis and pulmonary hypertension.^113^ Detailed spatial analyses of vascular hemodynamics will be feasible with 4D (i.e., three spatial dimensions and time‐resolved) flow MRI during exercise at 3 Tesla,^114^ which was recently used to visualize altered vortices in diastolic RV filling in preterm‐born but otherwise apparently healthy adolescents (*n* = 16) and adults (*n* = 10).^115^

Flow measurements of the great vessels allow for a quantitative evaluation of flow distribution and any changes therein during exercise.^28^ This is particularly relevant in patients with a Fontan circulation, who can suffer from exercise intolerance. Pedersen et al used 2D phase‐contrast MRI in pediatric patients to show that the increased flow from the inferior vena cava remained equally distributed to both lungs immediately after exercise, indicating that vascular resistance rather than geometry is a major determinant of pulmonary flow after total cavopulmonary connection surgery.^116^ Later, real‐time (i.e., without ECG triggering) 2D phase‐contrast MRI of different imaging planes enabled the assessment of flow in the aorta, the inferior and the superior vena cava in a free‐breathing regime *during* steady‐state supine bicycling exercise.^117^ Detailed analyses of the complex physiology during inspiration, expiration, and during exercise revealed how inspiration drives blood flow in the inferior vena cava at rest, but not during supine leg exercise when peripheral muscle contraction appears to be an important contributor to driving the circulation in those patients. Using a similar approach by acquiring 2D phase‐contrast MRI data of several abdominal vessels, the complex and differential hemodynamic responses to moderate‐intensity exercise in patients with abdominal aortic aneurysms and with peripheral artery disease were investigated.^19^

By measuring flow in the ascending aorta and pulmonary artery during exercise,^104^, ^118^ respective surrogate estimates of left ventricular (LV) and RV stroke volumes can be obtained.^119^, ^120^ This way, cardiac output can quantified without the need for acquiring multiple ventricular cine MRI series.^121^ Phase‐contrast MRI following exercise stress testing revealed a stiffer aortic wall as well as a blunted exercise‐induced augmentation of LV stroke volume in pulmonary hypertension compared with healthy subjects. This lack of stroke volume reserve was compensated for by a higher heart rate in order to maintain cardiac output.^122^ Real‐time 2D phase‐contrast MRI of blood flow in the ascending aorta has also been used to benchmark stroke volume estimates based on short‐axis cine MR imaging during exercise.^123^ Note that stroke volume estimates based on blood flow in these great vessels do not necessarily reflect the volumetric change of the ventricles, particularly in some scenarios where exercise intolerance is an important phenotype, eg, due to valve disease^124^ or conditions that cause shunting.

### Cardiac Function

Cardiac morphology and ventricular function is commonly quantified by multiplying the segmented surface areas of the ventricular blood pools and myocardial wall with the slice thickness and optional slice gap to obtain systolic and diastolic volumes and derived parameters^120^ from a stack of contiguous LV short‐axis 2D cine MRI series. Such datasets are acquired under breath‐hold conditions at rest over multiple heart beats using (retrospective) ECG gating^106^ in order to achieve the spatial and temporal alignment of the heart in contiguous and consecutive images that is important for accurate volumetry. In‐magnet physical exercise inherently comes with body movement and increased respiration, and the ECG signal becomes unreliable due to distortions by movement of the torso through the static magnetic field, potential heart rate fluctuations, and perspiration. These aspects preclude obtaining cine MRI series with ECG gating^106^ during exercise. Studies have tried to remedy these issues by acquiring data during breath holds in brief intervals of exercise cessation.^22^, ^125^ Such breath holds are difficult to perform, particularly by patients or with high‐intensity exercise, although cine MRI has even been performed with breath holds during exercise in healthy young adults.^126^

Acquisitions under free breathing conditions during exercise have been performed with “real‐time” MRI^127^ in order to visualize dynamic processes such as myocardial contraction and relaxation. Using radial *k*‐space undersampling and sensitivity encoding (SENSE) for parallel imaging in a radial *k*‐*t* SENSE sequence, Lurz et al acquired real‐time short‐axis cine MR images at a temporal resolution of 35 msec during various levels of steady‐state pedaling exercise (heart rates up to 154 ± 13 beats/min) without breath holds.^128^ The biventricular response to supine exercise in healthy volunteers was visualized, showing augmented LV and RV stroke volumes through a reduction in end‐systolic volumes (ESV) rather than an increase in end‐diastolic volumes (EDV); observations that were later confirmed in a meta‐analysis of similar studies.^126^ Although real‐time MRI mitigates movement disturbances and gating issues for each image, it typically generates a large number of images during minutes of data acquisition that still need to be spatially and temporally aligned according to their respiratory and cardiac phase for volumetric quantifications. These processing steps have been performed visually^128^, ^129^ or with the aid of separately recorded respiratory and ECG signals,^130^ but are labor intensive even if respiratory motion is ignored.^131^ One approach to reduce this processing burden is to lower the number of different imaging planes.^120^ Rather than acquiring a stack of contiguous short‐axis slices covering the heart, orthogonal long‐axis planes can be used to estimate (left) ventricular volumes during exercise.^126^, ^132^ Recent efforts have focused on (semi‐)automating the processing of real‐time MR images, eg, through principal component analysis of image signal intensities to extract the respiratory signal,^133^, ^134^ or based on periodic signal intensity fluctuations across the liver‐lung interface as a retrospectively applied respiratory “navigator”.^123^ Nonetheless, to capture end‐systolic and end‐diastolic phases at high heart rates, MRI at high temporal resolution is needed, because low temporal resolution leads to underestimations of cardiac performance.^135^ Accelerated image acquisition and increased temporal resolution (<30 msec) have been achieved through radial imaging,^136^ providing retrospective flexibility in temporal resolution during image reconstructions.^137^

Building on their approach to quantify biventricular volumes during high‐intensity bicycling exercise at 1.5 Tesla,^130^ La Gerche et al have since conducted a series of studies in various populations.^107^ In endurance athletes, the occurrence of ventricular arrhythmias may be related to the large hemodynamic load put on the RV during high‐intensity exercise, inducing transient RV dysfunction and potentially chronic RV remodeling. By utilizing the good visibility of both ventricles on MRI, it was demonstrated that in athletes with arrhythmias (*n* = 9), RV contractile dysfunction arose during exercise stress (i.e., attenuated reduction in RV ESV), while cardiac function appeared normal at rest.^138^ Cardiac dysfunction and reduced exercise capacity are potential adverse consequences of anthracycline‐based chemotherapy in breast cancer. In a randomized clinical trial investigating the impact of a 12‐month exercise training program on cardiorespiratory fitness in women (*n* = 102) with breast cancer who received such chemotherapy, MRI‐estimated cardiac reserve (i.e., exercise‐induced changes in stroke volumes, cardiac output, ejection fractions) served as a quantitative outcome measure.^139^ Exercise training improved cardiac reserve, while cardiac reserve declined in the usual‐care arm of the study, providing direct evidence that exercise training during anthracycline‐based chemotherapy can prevent cardiac dysfunction. Moreover, changes in cardiac reserve were mirrored by improvement or decline of VO_2_max, respectively. Such studies highlight the quantitative sensitivity of MRI‐based volumetry during exercise, and how its noninvasive nature readily allows for longitudinal measurements in patients as well as healthy volunteers. Many other examples of applications for ventricular volumetry during exercise, such as in patients with a Fontan circulation^140^ or repaired Tetralogy of Fallot,^129^ have been reviewed elsewhere.^107^

**FIGURE 10:**
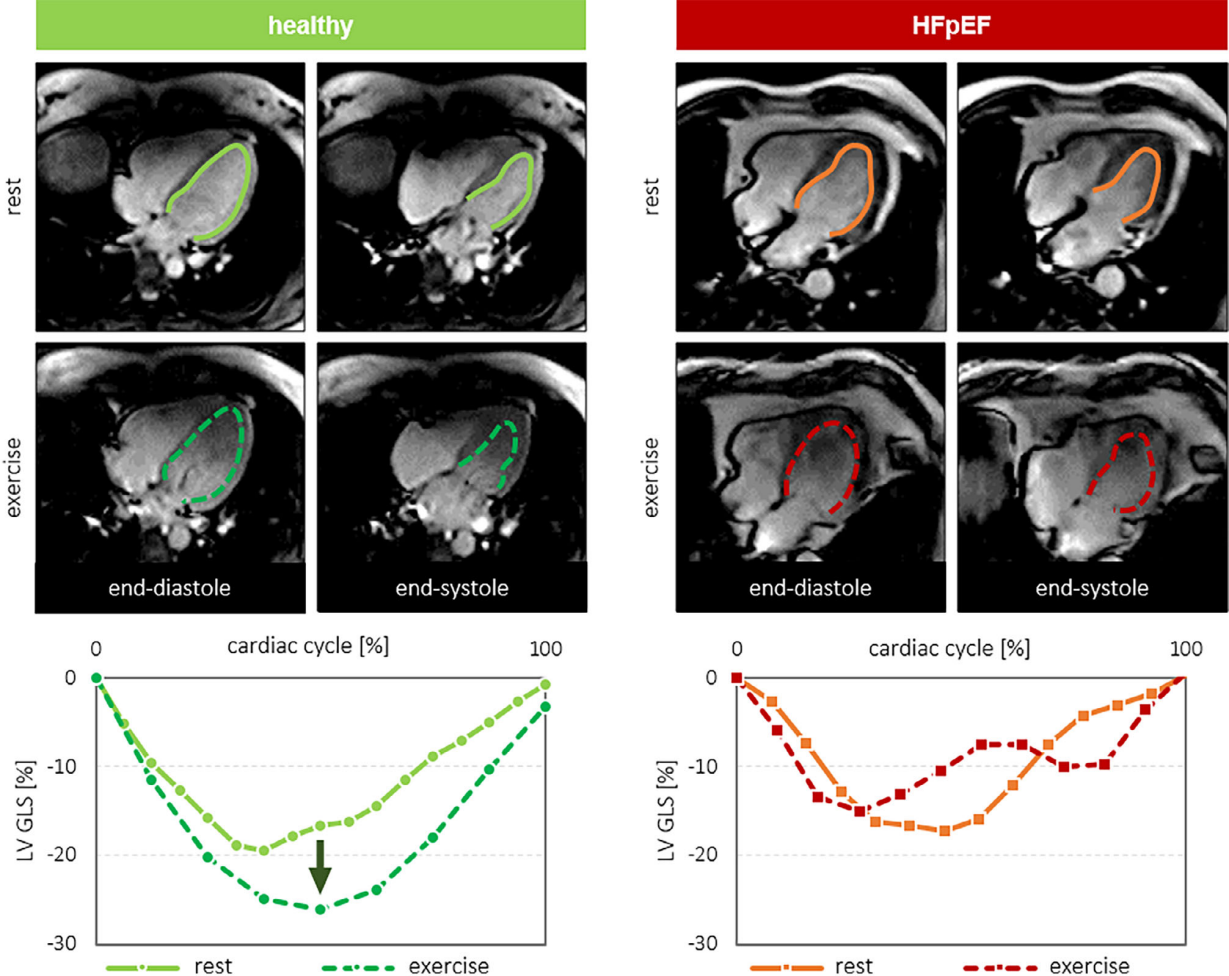
Example of how real‐time cine MRI during supine bicycling exercise stress can unmask myocardial dysfunction. Note that left ventricular (LV) global longitudinal strain (GLS) measured along the endocardial LV wall is augmented (green arrow) upon exercise in the healthy subject (male; 28 years). In the patient (male; 70 years) with heart failure with preserved ejection fraction (HFpEF), LV GLS appears normal at rest (orange), but fails to increase during exercise (red). Data courtesy of the Department of Radiology and Nuclear Medicine, Amsterdam University Medical Centers, Amsterdam, The Netherlands.

Assessments of myocardial strain can serve as measures of myocardial deformation and (dys)function beyond volumetric analyses, particularly through quantifications based on feature tracking of the endocardial walls in cine MRI.^135^, ^141^ Global longitudinal strain (GLS) can be assessed from long‐axis views and is sensitive to changes in contractility, with more negative values indicating higher contractility (Fig. 10). With exercise, normal LV GLS^132^ and RV GLS^142^ decrease from approximately −20% at rest to nearly −30%, while this exercise‐induced decrease is attenuated with age^132^ and in the RV of patients with pulmonary hypertension,^142^ indicating reduced contractile reserve. As an alternative to MRI feature tracking, others have estimated LV long‐axis strain by manually measuring the distance between the mitral valve and the apex in end‐diastolic and end‐systolic images.^143^ This approach avoids elaborate processing steps to obtain the complete cardiac cycle for feature tracking of the myocardial walls, particularly with real‐time MRI during exercise. Backhaus et al used supine bicycling exercise at 3 Tesla in a clinical trial to establish the accuracy of long‐axis strain measurements for HFpEF diagnosis.^143^ Left atrial long‐axis strain derived from real‐time MRI correlated strongly with the pulmonary capillary wedge pressures (PCWP) measured invasively with right heart catheterization during exercise (*n* = 75, *r* = −0.75, *P* < 0.001) as an indicator of impaired diastolic emptying in HFpEF during exercise. As such, their study showed that real‐time MRI during physiological bicycling exercise can qualify as a noninvasive alternative to the current reference test of invasive right heart catheterizations for diagnosing HFpEF.

While volumetry and global strain analyses provide whole‐chamber functional assessments, more detailed evaluations of myocardial wall deformation can be obtained through MR tagging.^144^ Using a radial tag pattern, real‐time MRI at 3 Tesla was used to reveal substantial diastolic recoil of the LV during early filling in a volunteer (*n* = 1) at low‐intensity bicycling exercise.^145^ Note that although imaging was done in real‐time, breath holds and triggering to the ECG signal were still required to accurately apply the tag pattern. Recently, real‐time MRI with ECG‐triggered tag line preparation at a temporal resolution of 29 msec was introduced for use immediately after supine bicycling exercise, showing a blunted response of LV deformation to exercise in HFpEF.^146^ Assessments of the regional response of myocardial deformation to exercise may aid in the evaluation of functional consequences of microvascular dysfunction, that may otherwise remain undetected at rest.

### Myocardial Perfusion

In patients with suspected coronary artery disease, perfusion imaging is an important approach for diagnosis and risk stratification.^147^ Stress is then used to evoke myocardial ischemia, most commonly through intravenous infusion of a vasodilator (eg, adenosine) or alternatively with an inotropic stress agent (eg, dobutamine). During such pharmacological stress with MRI, the first pass perfusion of a bolus of an gadolinium‐based contrast agent is dynamically imaged, revealing any hypoperfused regions and perfusion defects as an indicator of inducible myocardial ischemia.^148^ While pharmacological stress testing is sensitive, highly reproducible, and allows the subject to lie still during image acquisition, it does not reflect the hemodynamic and neurohormonal response to exercise or the physical activity that may induce exertional symptoms in the patient's daily life. First attempts to address these limitations involved upright treadmill exercise adjacent to the MR system, with the subjects quickly transferred back to their original position inside the MR bore after exercise followed by image acquisition within 1 minute.^16^ In later work, an MR‐compatible ergometer was mounted on the MR table to reduce the transition time between exercise and imaging.^149^ Supine bicycling exercise was performed with the table moved out of the bore, after which the table was repositioned into the magnet for imaging of myocardial perfusion shortly after exercise. Accelerated MR imaging was achieved through a radial steady‐state free precession sequence in combination with respiratory motion tracking^149^ and advanced reconstruction methods for undersampled data,^150^ allowing for an evaluation of myocardial first pass perfusion under free‐breathing conditions. Measurements of first pass perfusion with MRI after treadmill exercise have shown diagnostic merit over treadmill stress testing with single photon emission computed tomography (SPECT) of patients (*n* = 210) with known or suspected coronary artery disease in a multi‐center clinical trial.^17^ In another clinical trial, the capacity of exercise MR stress testing to detect defects and regional wall motion abnormalities in patients (*n* = 60) with suspected coronary artery disease was benchmarked against invasive fractional flow reserve (FFR) measurements with angiography,^151^ again showing potential to diagnose coronary artery disease noninvasively. Note that although the intravenous administration of a pharmacological stress agent is no longer needed with physical exercise stress, first pass perfusion measurement still requires infusion of a gadolinium‐based contrast agent as a blood flow tracer.

**FIGURE 11:**
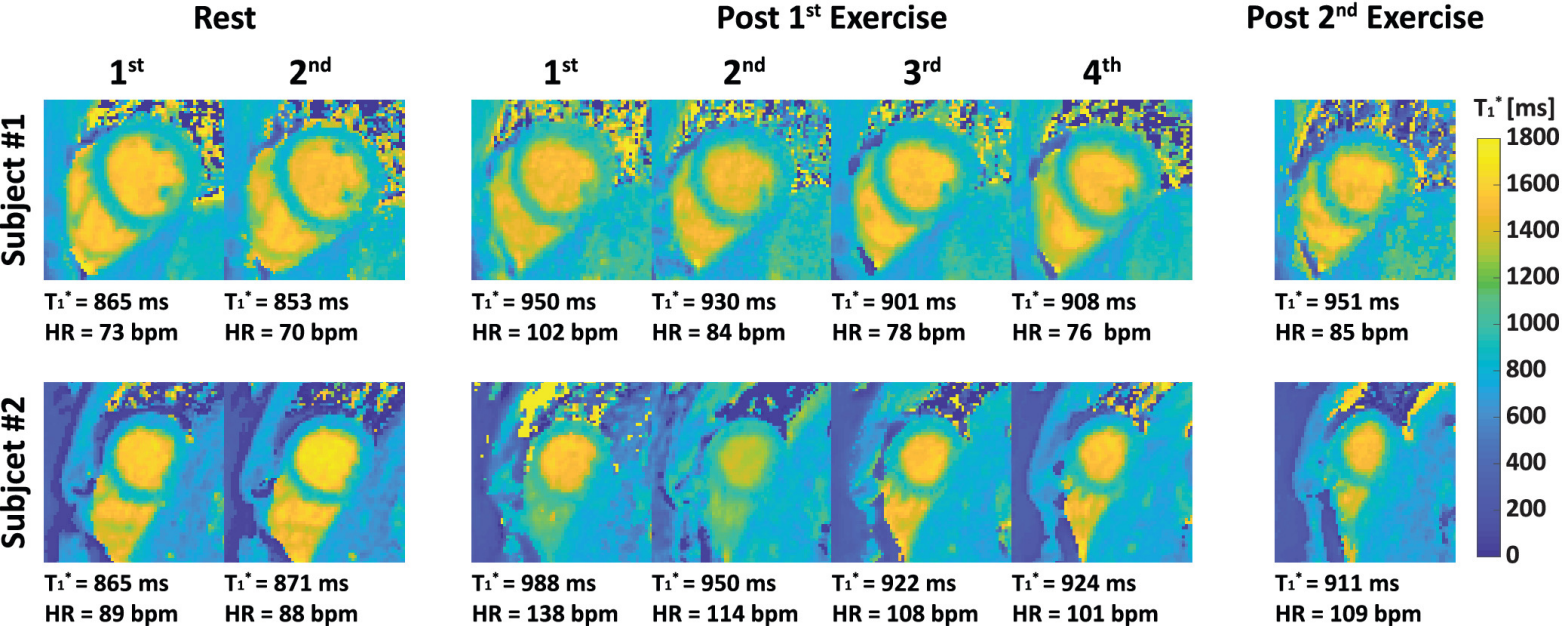
Maps of apparent myocardial T_1_ (T_1_*) values in two healthy volunteers at rest and after two bouts of vigorous supine bicycle exercise on an MR‐compatible ergometer mounted on the table of a 3 Tesla MR system. Note the increase and subsequent gradual decrease of myocardial T_1_* values post‐exercise, indicating an exercise‐induced elevation of myocardial perfusion. bpm = beats per minute; HR = heart rate. Reproduced from Guo et al^156^ with permission from John Wiley & Sons, Inc. © 2022 International Society for Magnetic Resonance in Medicine.

Changes in the myocardial native T_1_ relaxation time constant that occur with changes in myocardial blood flow or volume^152^ have been utilized for the detection of ischemia under adenosine stress without the need for a gadolinium‐based contrast agent.^153^ Following this concept, Nakamori et al then showed that myocardial native T_1_ is transiently elevated by 6 ± 3% within 30 s after supine bicycling exercise on an MR‐compatible ergometer mounted on the table of a 1.5 Tesla MR system.^154^ Importantly, the magnitude of T_1_ reactivity correlated (*n* = 28, *r* = 0.62, *P* < 0.001) with the maximum heart rate—blood pressure product, an index of myocardial oxygen consumption during exercise.^155^ Those data suggest that native T_1_ mapping can provide a surrogate marker of any physiological change in myocardial blood flow during exercise. Indeed, in patients (*n* = 14) with coronary artery disease, native T_1_ reactivity to exercise mirrored the severity of myocardial perfusion abnormalities confirmed with SPECT.^154^ Using a “free‐running” radial spoiled gradient echo sequence with respiratory navigator readouts and retrospective ECG gating,^156^ exercise‐induced T_1_ reactivity of +5% to +10% in healthy volunteers was measured at 3 Tesla (Fig. 11). Importantly, all studies with spatial information on myocardial perfusion, either through first pass perfusion imaging or with T_1_ parameter mapping, acquired their data quickly *after* exercise. Such measurements *during* physical exercise have not yet been demonstrated. Challenges to do so involve the high heart rate on top of fast and irregular or deep respiration, as well as potential upper body movement during leg exercise, all compromising the alignment of consecutive images that is required for quantitative MR of the myocardium, while the exercise protocol needs to be performed *inside* the MR system for simultaneous measurements. With low‐intensity isometric hand grip exercise that allows in‐magnet exercise without body movement, an increase (+50.7 ± 31.4%) in peak coronary blood flow during was measured with phase‐contrast MRI in the left anterior descending coronary artery of normal volunteers (*n* = 9) at 1.5 Tesla.^157^ This approach was later used at 3 Tesla to unmask a reduction in coronary flow in patients with coronary artery disease, demonstrating the ability to assess coronary endothelial (dys)function noninvasively.^158^

### Myocardial Energy Metabolism

Nearly four decades ago, among the first applications of MR to study the human heart was measuring phosphate metabolite levels in the in vivo myocardium with ^31^P‐MRS.^159^ It was soon realized that such “*measurements at rest did not clearly differentiate between normal and diseased myocardium*,”^160^ and that studies during exercise would be needed. Today, essentially all ^31^P‐MRS studies follow the pioneering work from the early 1990s,^161^, ^162^, ^163^ and assess myocardial high‐energy phosphate metabolism by quantifying the PCr over ATP signal ratio as a measure of the in vivo myocardial energy status, while the signal from myocardial P_i_ cannot be resolved. Because ^31^P‐MRS of the heart requires multiple transients to achieve a sufficient signal‐to‐noise ratio as well as spatial localization (eg, with a multi‐shot 3D image‐selected in vivo spectroscopy [ISIS] sequence^164^), such acquisitions are lengthy (i.e., multiple minutes) relative to single‐shot dynamic ^31^P‐MRS acquisitions in skeletal muscle (temporal resolution of seconds, see above). This precludes any *dynamic* detection of how the PCr/ATP ratio changes during transitions between cardiac work rates. Myocardial PCr/ATP ratios therefore only inform on *steady‐state* conditions of myocardial energy and proton balance, and are measured during submaximal exercise only.

**FIGURE 12:**
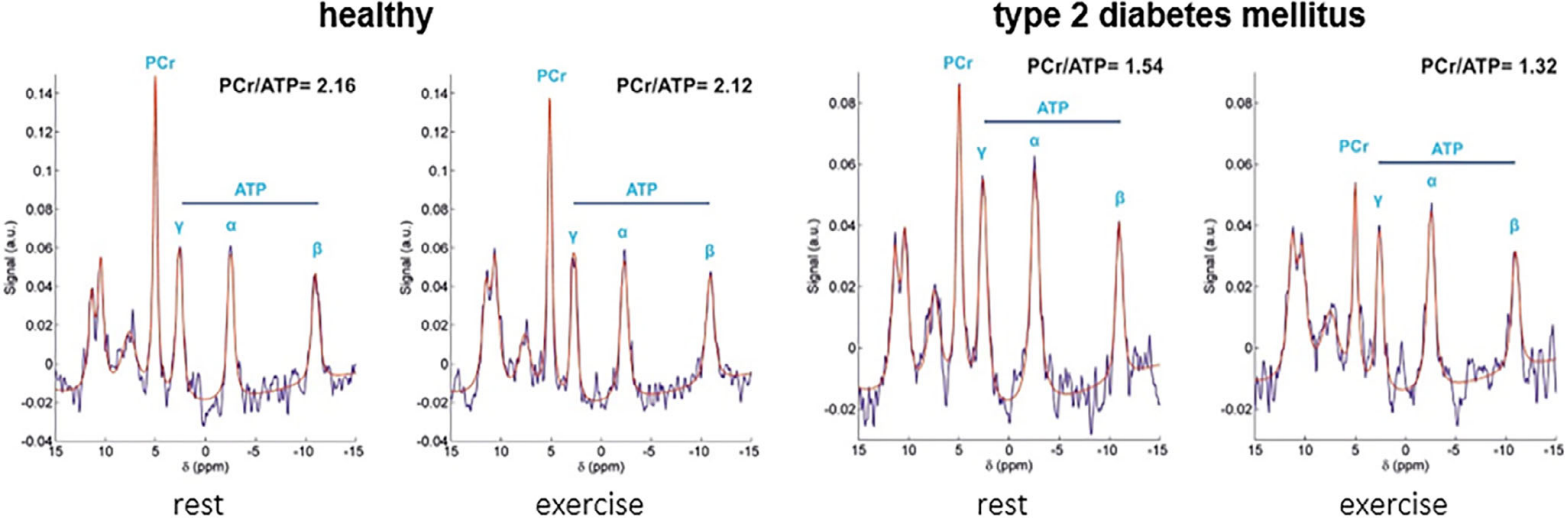
Phosphorus‐31 MR spectra (^31^P‐MRS) acquired at rest and during steady‐state prone repeated and alternate knee flexion exercise inside a 3 Tesla MR system in a healthy subject and a patient with type 2 diabetes mellitus. Note the low myocardial PCr/ATP ratio in type 2 diabetes mellitus at rest that decreases further upon moderate‐intensity exercise, indicating impaired myocardial energy homeostasis. α, β, γ‐ATP = α‐, β‐, γ‐phosphate groups in adenosine 5′‐triphosphate; PCr = phosphocreatine. Reproduced and rearranged with adapted labels from original by Levelt et al^171^ under a Creative Commons BY license (https://creativecommons.org/licenses/by/4.0/).

Given these methodological challenges of cardiac ^31^P‐MRS, a change of the myocardial PCr/ATP ratio during exercise or recovery in healthy subjects has not been detected with ^31^P‐MRS. Most studies have been conducted at low‐ or moderate‐intensity exercise, such as isometric hand gripping^161^ or prone knee flexion.^160^ Moreover, measurement variability increases substantially during exercise,^165^ further degrading the sensitivity for detecting any exercise‐induced reduction of the myocardial PCr/ATP ratio that computational modeling estimated to be maximally <10% for healthy subjects at maximal exercise intensity.^166^ In contrast, in various patient populations, localized ^31^P‐MRS of the heart has successfully been applied during exercise to expose impaired myocardial energy homeostasis. Using a 1.5 Tesla MR system, Weiss et al showed that the PCr/ATP ratio of the anterior myocardium in patients (*n* = 16) with coronary artery disease transiently decreased (−37%) from 1.45 ± 0.31 at rest (77 ± 13 beats/min) to 0.9 ± 0.24 during isometric hand grip exercise (89 ± 16 beats/min)^161^; observations that were later replicated by others.^167^, ^168^ Moreover, revascularization therapy restored the impaired myocardial energy homeostasis that was observed during exercise.^161^ In patients (*n* = 35) with hypertrophic cardiomyopathy (HCM) due to a pathogenic mutation, ^31^P‐MRS revealed a reduced myocardial energy status at rest that exacerbated upon dynamic prone knee flexion (90 ± 12 beats/min).^169^ This observation supports the notion that inefficient mechanical function due to sarcomere mutations increases the myocardial energy *demand* for maintaining adequate cardiac pumping performance.^170^ In a scenario energy *supply* is compromised, Levelt et al showed (Fig. 12) that myocardial PCr/ATP was 17% lower in type 2 diabetes mellitus patients (*n* = 31) at rest, which further decreased by 12% during moderate‐intensity exercise (69 ± 8 beats/min), attributed to hypoperfusion that was demonstrated separately with BOLD‐MRI under adenosine stress.^171^ These studies show how noninvasive measurements of the in vivo myocardial energy status during exercise can inform on myocardial pathology.

Not surprisingly, exercise ^31^P‐MRS of the heart has not yet been translated to the clinic. Measurements are lengthy, prone to (motion) artifacts and suffer from poor sensitivity, and require dedicated hardware and specific expertise that is not typically available. There are large differences in reported PCr/ATP ratios between studies and between sites,^166^ and reference values are lacking. More recent efforts have focused on reducing acquisition times for higher temporal resolution and improving measurement sensitivity and precision by using 7 Tesla MR systems.^172^, ^173^, ^174^ Using single‐shot single‐voxel stimulated echo acquisition mode (STEAM) localization to suppress the 2,3‐diphosphoglycerate (2,3‐DPG) signal in flowing ventricular blood, the detection of myocardial P_i_ has been reported.^175^ If confirmed, this would allow for noninvasive estimations of myocardial pH changes.^176^ Moreover, a rise in P_i_ concentration may be a more sensitive marker of impaired myocardial energy homeostasis than the PCr/ATP ratio.^166^ Yet, a first study in healthy subjects (*n* = 17) under dobutamine stress did not detect any change in myocardial P_i_ or pH.^175^ Any ^31^P‐MRS measurements of the human heart during exercise in a 7 Tesla MR system have yet to be reported.

## Physical Exercise and the Brain

With very limited intracellular stores of energy substrates, the brain is reliant on an adequate and continuous supply of nutrients and oxygen. An adaptive cerebrovascular system that can cope with fluctuations in supply and demand during physiological challenges is therefore crucial to maintain healthy brain function. To assess cerebrovascular health with MR, typically hypercapnic (inhalation of 5% CO_2_)^177^ or pharmacological (intravenous infusion of acetazolamide)^178^ stimuli are used to elicit a maximal vasodilatory response such that the cerebrovascular reserve capacity relative to baseline conditions can be estimated. Such laboratory‐controlled stimuli do not reflect daily life conditions. Instead, physical exercise is a physiologically relevant challenge^179^ that has been applied in MR investigations of the human brain, albeit scarcely.

Until recently, imaging studies of the human brain during exercise^180^ were limited to utilizing the accessibility of the middle cerebral artery (MCA) for ultrasound imaging through the temporal acoustic window.^181^ By measuring the blood flow velocity with transcranial Doppler ultrasound, the cerebral blood flow (CBF) can be estimated under the assumption of a constant vessel diameter. Using a 7 Tesla MR system to obtain black‐blood images at a high in‐plane spatial resolution of 0.2 × 0.2 mm^2^, Verbree et al showed that the cross‐sectional area of the MCA lumen decreased by 2.1 ± 0.8% during rhythmic hand grip exercise.^182^ Although exercise intensity was very low and induced only a very mild 11.2 ± 1.7% increase in heart rate to 68.4 ± 12.5 beats/min, the high spatial resolution at 7 Tesla provided sufficient sensitivity to detect the minute vasoconstriction of the MCA due to exercise‐induced sympathetic activation. Those data suggest that assuming a constant vessel diameter may lead to an overestimation of the increase in CBF with transcranial Doppler ultrasound during low‐intensity exercise. Using several different imaging planes for 2D phase‐contrast measurements at 3 Tesla, Tarumi et al made a detailed analysis of the interplay between CBF and cerebrospinal fluid (CSF) flow during low‐intensity rhythmic hand grip exercise, showing how both CBF and CSF flow are coupled to maintain intracranial volume‐pressure homeostasis in healthy volunteers.^183^

**FIGURE 13:**
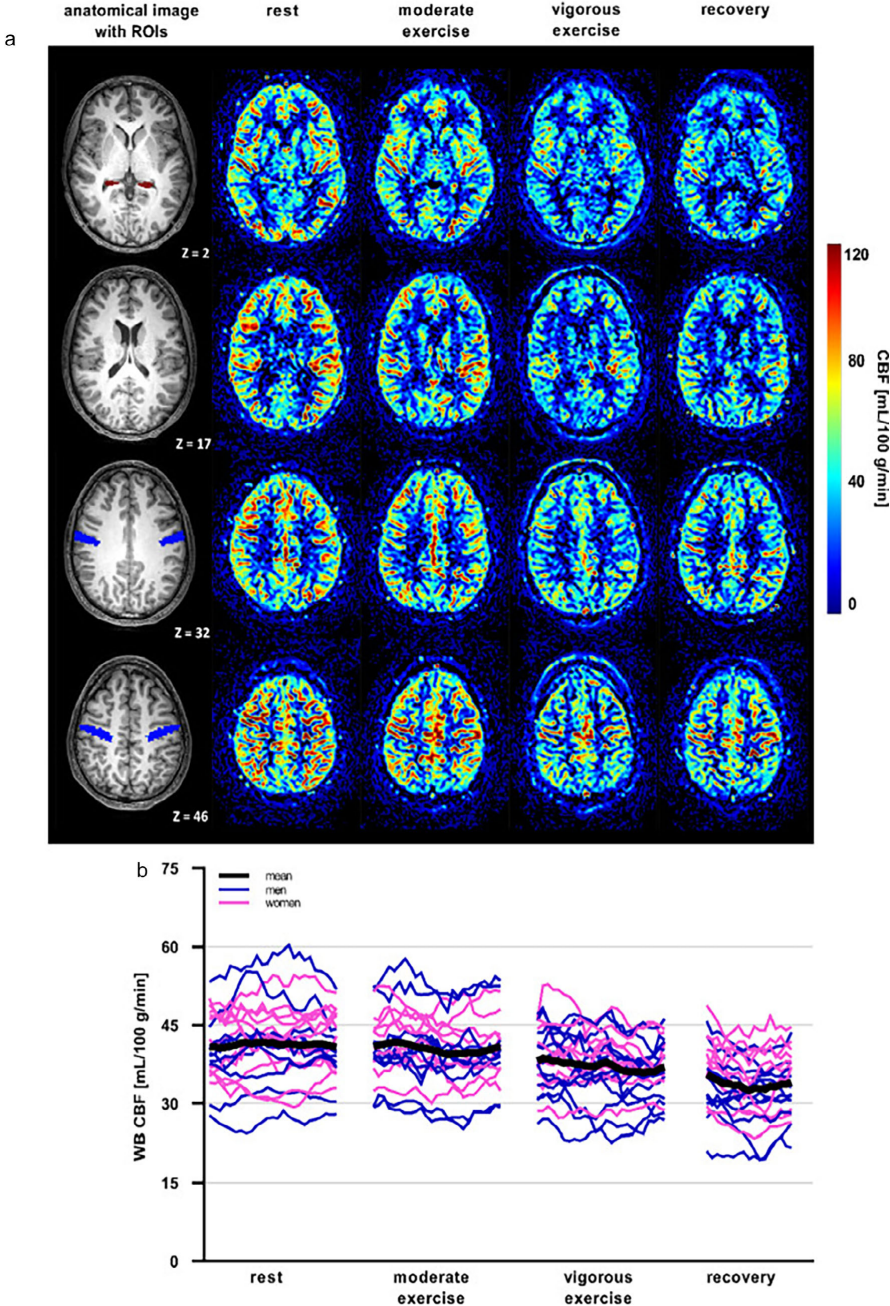
Quantitative maps of cerebral blood flow (CBF) obtained with pseudo‐continuous arterial spin labeling (pCASL) MRI at 3 Tesla at rest, during various stages of steady‐state supine bicycling exercise, and during subsequent recovery (**a**). Regions of interest for hippocampus and motor cortex are outlined in red and blue, respectively, on T_1_‐weighted anatomical images with the MNI‐space (Montreal Neurological Institute, Montreal, Quebec, Canada) z‐coordinate indicated. Note the enhanced perfusion of the skin during exercise and subsequent recovery. Time curves (temporal resolution, 9.1 s per control/label pair) of the mean whole‐brain (WB) CBF (**b**) show a consistent decrease during vigorous exercise that continued during recovery. Reproduced from Mast et al^185^ with permission from Elsevier under a Creative Commons BY‐NC‐ND license (https://creativecommons.org/licenses/by‐nc‐nd/4.0/).

Estimating CBF from the blood flow in a single large cerebral vessel is a rather coarse method for assessing whole‐brain behavior, because any differences between brain regions cannot be resolved. ASL‐MRI^184^ provides quantitative maps of tissue perfusion using magnetically labeled inflowing blood as an endogenous “tracer”.^68^ Based on the subtraction of control‐ and label‐images, ASL‐MRI is deemed to be susceptible to motion‐induced errors. Nonetheless, Mast et al demonstrated that it is feasible to collect quantitative data on cerebral perfusion of the whole brain as well as its different regions using pseudo‐continuous ASL (pCASL) MRI during bicycling exercise in a 3 Tesla MR system.^185^ At a spatial resolution of 2.75 × 2.75 × 5 mm^3^ and a temporal resolution of 9.1 s per control/label pair, pCASL datasets were acquired at rest, during 5 minutes of steady‐state exercise at moderate intensity with heart rates of ~100 beats/min and at vigorous intensity (~140 beats/min), and during initial recovery. Whole‐brain CBF remained similar during moderate exercise, decreased by >10% during vigorous exercise, and further decreased during initial recovery by 15% compared with resting‐state CBF (Fig. 13). Strikingly different from whole‐brain behavior, the motor cortex CBF increased by >12% during moderate exercise, returned to resting‐state values during vigorous exercise, and decreased by >17% during recovery. This downward parabolic curve of motor cortex CBF relative to exercise intensity agrees with studies of cerebral perfusion during exercise^180^ that used ultrasound measurements of the MCA. Indeed, the motor cortex is fed predominantly by the MCA, and its perfusion may differ from other brain regions during physical exercise as was shown with positron emission tomography (PET)^186^ and now also with MRI.^185^ In the latter study, whole‐brain CBF measurements with pCASL‐MRI were benchmarked against estimates based on 2D phase‐contrast measurements of blood flow in the common carotid arteries. Those large‐vessel surrogate measures overestimated whole‐brain CBF by >30 mL/100 g/min, which proportionally increased for higher CBF values. This became most apparent during vigorous exercise, with a large proportion of the blood flow in the carotid arteries directed through the external carotid artery toward the skin for thermoregulation. Indeed, elevated skin perfusion became apparent in pCASL perfusion maps as enhanced perfusion‐weighted signal in the scalp (Fig. 13). Interestingly, that study reported a lower cerebral metabolic rate of oxygen after vigorous‐intensity exercise, which suggests that brain oxygen demand during recovery was lower than during resting‐state conditions.^185^ It is intriguing to consider that such reduced brain oxygenation may be a mediator for the sensation of fatigue acting as a limiting factor for exercise capacity, but data to support this notion are currently lacking.

Several studies have used MRI to investigate the brain immediately *after* a single bout of exercise, i.e., during recovery. A small pioneering study in young healthy adults (24.8 ± 1.5 years; *n* = 5) showed that whole‐brain CBF measured with pulsed ASL at 3 Tesla was approximately 20% higher 10 minutes into recovery after a 30‐minute bout of moderate‐intensity (125 beats/min) upright bicycling exercise.^187^ Given the notion that habitual physical activity may improve hippocampal structure and function,^188^ regional CBF after exercise has been investigated for the hippocampus in healthy adults, showing either a ~20%^189^ up to 30%^185^ reduction or a >10%^190^ elevation of perfusion. Another study in older subjects (70.7 ± 3.1 years; *n* = 25) showed that lower hippocampal CBF at 7 minutes after exercise was associated with higher cognitive performance.^191^ In stroke survivors, pCASL MRI after 20 minutes of low‐intensity (100 beats/min) or moderate‐intensity (110 beats/min) semi‐recumbent bicycling exercise revealed region‐specific and exercise intensity‐dependent changes in CBF, with low perfusion in ischemic lesions that did not change after exercise.^192^ Others have used BOLD‐MRI to map cardiac‐related brain pulsatility in response to exercise,^193^ which could serve as a measure of arterial stiffness when evaluating cerebrovascular health.^194^ More research on such acute effects of exercise on the brain is warranted in order to better understand the mechanisms of how physical exercise can be beneficial for cerebrovascular health and cognitive performance,^195^ eg, in aging^13^ or in stroke rehabilitation.^196^

## Outlook

Exercise MR stress testing provides a paradigm for noninvasive assessments of human (patho)physiology. This methodology is noninvasive and nonionizing, and therefore very suitable for (repeated) measurements in healthy volunteers as well as patients. Yet, despite ample demonstration of its feasibility and merits in various scenarios and patient populations, application of exercise MR has predominantly been restricted to studies of relatively small cohorts in a research setting. Broadly accepted protocols for use in a clinical workflow are currently lacking. Practical barriers remain the technological complexities associated with obtaining meaningful quantitative MR readouts during exercise, which require local expertise, dedicated hardware such as suitable RF coils and MR‐compatible ergometers, and typically dedicated software for data processing and analysis. Performing exercise within the confined space of an MR scanner bore while fitted with vital sign sensors, RF coils and an ergometer may be daunting for subjects. On the other hand, the subject remains in control of the self‐applied stress intensity, and may halt exercise instantaneously, with infusion of pharmacological stress agents and catheterizations for pressure measurements no longer required.

With continued technological advances, we foresee improvements of various aspects of exercise MR stress testing. Vendors have put substantial effort into providing authority‐approved equipment and methods for non‐proton (eg, phosphorus‐31) MR evaluations. However, the frequent use of custom‐built ergometer equipment for exercise MR stress testing, particularly for musculoskeletal applications, hamper authority approval for their clinical use. Strong collaboration and coordination between MR ergometer users and vendors is needed to implement authority‐approved devices that are validated for multiple body parts and for broad‐scale clinical applications. With so‐called “interleaved” scanning of several different acquisitions, eg, ^31^P‐MRS and BOLD ^1^H‐MRI,^197^ multi‐parametric MR readouts can be obtained essentially simultaneously. This concept is ideal for exercise stress testing, which is typically limited to a *single* session that is very difficult to replicate precisely, and paves the way for a more integrative approach to study muscle oxygen utilization and ATP turnover.^81^ Such integrative approaches are particularly important for evaluating exercise intolerance and fatigue, where the underlying mechanisms could be misinterpreted when based solely on individual MR readouts. Alternatively, other noninvasive techniques to investigate skeletal muscle physiology such as surface electromyography^198^ and NIRS^199^ may be integrated into exercise MR stress testing examinations, amplifying its clinical utility.

Advancements in machine learning will contribute to pushing the limits of temporal and spatial resolution for MRI during exercise,^200^ such that dynamic physiological processes can be captured and analyzed in even more detail. For instance, evaluations of ^31^P‐MRS data in skeletal muscle have predominantly focused on quantifying the PCr recovery rate from series of spectra obtained during recovery *after* exercise, mostly for practical reasons and because the PCr resonance frequency is robust to any pH changes.^31^ In contrast, extracting phenotypical information from series of spectra acquired *during* exercise is much less straightforward, particularly if the exercise mode or intensity recruits white myofibers along with red and intermediate myofibers (Fig. 3). Analyses of such series are labor intensive and sensitive to user input, and therefore represent a bottleneck in the processing workflow, which precludes swift automated evaluation, and thus stalling routine clinical use of skeletal muscle exercise ^31^P‐MRS stress testing. The application of neural networks that capture the known mechanistic biochemical and physiological correlations between state variables in time may render the processing and quantitative analysis of such datasets amenable for automation. Moreover, machine learning can help in overcoming the labor‐intensive burden of image processing and segmentation^201^ for quantification of cardiac function during exercise MR stress testing, improving its applicability in the clinical workflow. Indeed, commercial software packages for cardiac function analysis already include machine learning algorithms to generate ventricular contours automatically. While trained to segment conventional cine MRI series of the heart, neural networks provided automated segmentations the LV and RV volumes in real‐time MRI series at rest with an accuracy that is similar to the inter‐observer variability for manual segmentation.^202^ Although promising, the performance of those deep learning methods is not yet sufficient for a fully automated analysis of real‐time MRI series acquired during exercise.^202^ Using data from a free‐breathing multi‐parametric mapping sequence, a neural network can be used to substantially reduce the time‐consuming image reconstruction process, by reconstructing myocardial T_1_ and T_2_ maps nearly instantaneously.^203^ Its applicability to exercise MR stress testing, eg, for probing myocardial perfusion with T_1_ mapping^156^ or blood oxygenation with T_2_ mapping,^204^ still needs to be demonstrated. Advanced machine learning approaches increasingly find their way in many aspects of MR examination^201^ such as data acquisition, image reconstruction, and quantitative analysis. We foresee that exercise MR, which typically requires fast image acquisitions, generates large datasets, and potentially suffers from low signal‐to‐noise ratios and motion artifacts, will particularly benefit from the maturation of machine learning.

Driven by advancements in data acquisition and reconstruction techniques, applications of low magnetic field (i.e., ~0.5 Tesla) MR systems have regained interest.^205^ For instance, MRI at low magnetic field allows imaging of the lungs due to its superior magnetic field homogeneity that reduces susceptibility artifacts near air‐tissue interfaces. Indeed, Seeman et al used supine pedaling exercise in clinical MR system ramped down to 0.55 Tesla to demonstrate dynamic accumulation of lung water after vigorous‐intensity exercise and its subsequent clearance in healthy volunteers (*n* = 15) and patients with heart failure (*n* = 2).^206^ Such measurements of lung water could aid in examinations of (exercise‐induced) transient pulmonary congestion in HFpEF.^207^ Additionally, the low magnetic field reduces motion‐induced distortions of the ECG signal, may alleviate some of the constraints on the design and construction of ergometers and auxiliary equipment such as blood pressure monitors, and more readily affords the design of novel MR systems with a vertical open‐bore configuration.^205^ Open‐bore MR systems offer more space to perform physical exercise, and allow for evaluations of the musculoskeletal system under weight‐bearing conditions. Moreover, such setups may open up opportunities to revive past approaches for upright exercise MR stress testing^18^ for a more integrated, systemic investigation of exercise physiology.^19^, ^208^

The soft tissue contrast of MRI provides noninvasive access to essentially all organs of the human body. As such, it allows for an integrated evaluation of peripheral and central function beyond an individual organ. Indeed, a recent study used ^31^P‐MRS of PCr recovery kinetics to link better mitochondrial function in skeletal muscle with a reduced risk of mild cognitive decline and dementia, suggesting that mitochondrial dysfunction may play an important role in Alzheimer's disease.^209^ Whereas early in vivo ^31^P‐MRS measurements during mechanical tasks that involved single limb muscles revealed profound intramuscular acidification,^75^ studies of skeletal muscle energy homeostasis and pH balance during exhaustive two‐legged bicycling exercise showed a dampened intramuscular pH drop.^25^, ^210^ This phenomenon was attributed to the superior cardiovascular and respiratory support during exercise that recruits a large muscle mass relative to single‐limb exercise.^210^ Such measurements could be extended to investigate the systemic effects of whole‐body exercise on other organs, such as the brain.^211^ Moreover, the impact of any disease that affects cardiovascular or pulmonary function on peripheral organs that rely heavily on adequate substrate supply and removal of metabolic waste products can be quantitatively assessed with exercise MR stress testing,^25^ potentially serving to inform therapeutic strategies.

Despite many advancements in the past decades, exercise MR arguably still represents a niche area in the tremendously versatile field of MR. It is up to our community of MR researchers, clinicians and physiologists to keep moving forward by providing innovative solutions and meaningful examples. At the same time, we advocate for continued support from vendors of MR systems, processing and analysis software packages, and ergometers by ensuring compatibility of their tools and equipment with exercise MR stress testing. Such joint efforts are warranted to consolidate exercise MR stress testing as a valuable contributor to our understanding of human exercise physiology and exercise intolerance.

## References

[1] Ross R , Blair SN , Arena R , et al. Importance of assessing cardiorespiratory fitness in clinical practice: A case for fitness as a clinical vital sign: A scientific statement from the American Heart Association. Circulation 2016;134:e653‐e699. 27881567 10.1161/CIR.0000000000000461

[2] Olpin SE . Pathophysiology of fatty acid oxidation disorders and resultant phenotypic variability. J Inherit Metab Dis 2013;36:645‐658. 23674167 10.1007/s10545-013-9611-5 PMC7101856

[3] Rapin A , Etossé A , Tambosco L , et al. Aerobic capacities and exercise tolerance in neuromuscular diseases: A descriptive study. Ann Phys Rehabil Med 2013;56:420‐433. 23669143 10.1016/j.rehab.2013.04.004

[4] Pandey A , Shah SJ , Butler J , et al. Exercise intolerance in older adults with heart failure with preserved ejection fraction: JACC state‐of‐the‐art review. J Am Coll Cardiol 2021;78:1166‐1187. 34503685 10.1016/j.jacc.2021.07.014 PMC8525886

[5] Brown JT , Saigal A , Karia N , et al. Ongoing exercise intolerance following COVID‐19: A magnetic resonance‐augmented cardiopulmonary exercise test study. J Am Heart Assoc 2022;11:e024207. 35470679 10.1161/JAHA.121.024207 PMC9238618

[6] Reusch JEB , Bridenstine M , Regensteiner JG . Type 2 diabetes mellitus and exercise impairment. Rev Endocr Metab Disord 2013;14:77‐86. 23299658 10.1007/s11154-012-9234-4 PMC3593997

[7] American Thoracic Society , American College of Chest Physicians . ATS/ACCP Statement on cardiopulmonary exercise testing. Am J Respir Crit Care Med 2003;167:211‐277. 12524257 10.1164/rccm.167.2.211

[8] Albouaini K , Egred M , Alahmar A , Wright DJ . Cardiopulmonary exercise testing and its application. Heart 2007;93:1285‐1292. 17890705 10.1136/hrt.2007.121558 PMC2000933

[9] Pieske B , Tschöpe C , de Boer RA , et al. How to diagnose heart failure with preserved ejection fraction: The HFA‐PEFF diagnostic algorithm: A consensus recommendation from the Heart Failure Association (HFA) of the European Society of Cardiology (ESC). Eur Heart J 2019;40:3297‐3317. 31504452 10.1093/eurheartj/ehz641

[10] Kithcart AP , Beckman JA . ACC/AHA versus ESC guidelines for diagnosis and management of peripheral artery disease: JACC guideline comparison. J Am Coll Cardiol 2018;72:2789‐2801. 30497565 10.1016/j.jacc.2018.09.041

[11] Distefano G , Goodpaster BH . Effects of exercise and aging on skeletal muscle. Cold Spring Harb Perspect Med 2018;8:a029785. 28432116 10.1101/cshperspect.a029785 PMC5830901

[12] Eijsvogels TMH , Molossi S , Lee D‐C , Emery MS , Thompson PD . Exercise at the extremes: The amount of exercise to reduce cardiovascular events. J Am Coll Cardiol 2016;67:316‐329. 26796398 10.1016/j.jacc.2015.11.034

[13] Bliss ES , Wong RH , Howe PR , Mills DE . Benefits of exercise training on cerebrovascular and cognitive function in ageing. J Cereb Blood Flow Metab 2021;41:447‐470. 32954902 10.1177/0271678X20957807 PMC7907999

[14] Chance B , Eleff S , Leigh JS . Noninvasive, nondestructive approaches to cell bioenergetics. Proc Natl Acad Sci U S A 1980;77:7430‐7434. 6938983 10.1073/pnas.77.12.7430 PMC350517

[15] Gadian DG , Radda GK , Dawson MJ , Wilkie DR . pH_i_ measurements of cardiac and skeletal muscle using ^31^P‐NMR. Kroc Found Ser 1981;15:61‐77. 6951958

[16] Jekic M , Foster EL , Ballinger MR , Raman SV , Simonetti OP . Cardiac function and myocardial perfusion immediately following maximal treadmill exercise inside the MRI room. J Cardiovasc Magn Reson 2008;10:3. 18272005 10.1186/1532-429X-10-3 PMC2244608

[17] Raman SV , Dickerson JA , Mazur W , et al. Diagnostic performance of treadmill exercise cardiac magnetic resonance: The prospective, multicenter exercise CMR's accuracy for cardiovascular stress testing (EXACT) trial. J Am Heart Assoc 2016;5:e003811. 27543308 10.1161/JAHA.116.003811 PMC5015300

[18] Cheng CP , Herfkens RJ , Taylor CA . Inferior vena caval hemodynamics quantified *in vivo* at rest and during cycling exercise using magnetic resonance imaging. Am J Physiol Heart Circ Physiol 2003;284:H1161‐H1167. 12595296 10.1152/ajpheart.00641.2002

[19] Tenforde AS , Cheng CP , Suh G‐Y , Herfkens RJ , Dalman RL , Taylor CA . Quantifying *in vivo* hemodynamic response to exercise in patients with intermittent claudication and abdominal aortic aneurysms using cine phase‐contrast MRI. J Magn Reson Imaging 2010;31:425‐429. 20099356 10.1002/jmri.22055 PMC2963312

[20] Schaefer S , Peshock RM , Parkey RW , Willerson JT . A new device for exercise MR imaging. AJR Am J Roentgenol 1986;147:1289‐1290. 3490765 10.2214/ajr.147.6.1289

[21] Jeneson JAL , Schmitz JPJ , Hilbers PAJ , Nicolay K . An MR‐compatible bicycle ergometer for in‐magnet whole‐body human exercise testing. Magn Reson Med 2010;63:257‐261. 19918886 10.1002/mrm.22179

[22] Gusso S , Salvador C , Hofman P , et al. Design and testing of an MRI‐compatible cycle ergometer for non‐invasive cardiac assessments during exercise. Biomed Eng Online 2012;11:13. 22423637 10.1186/1475-925X-11-13 PMC3334686

[23] Nyberg M , Jones AM . Matching of O_2_ utilization and O_2_ delivery in contracting skeletal muscle in health, aging, and heart failure. Front Physiol 2022;13:898395. 35774284 10.3389/fphys.2022.898395 PMC9237395

[24] Saltin B . Hemodynamic adaptations to exercise. Am J Cardiol 1985;55:42D‐47D. 10.1016/0002-9149(85)91054-9 3993550

[25] van Brussel M , van Oorschot JWM , Schmitz JPJ , et al. Muscle metabolic responses during dynamic in‐magnet exercise testing: A pilot study in children with an idiopathic inflammatory myopathy. Acad Radiol 2015;22:1443‐1448. 26259546 10.1016/j.acra.2015.06.013 PMC5449456

[26] Vegter RJK , van den Brink S , Mouton LJ , Sibeijn‐Kuiper A , van der Woude LHV , Jeneson JAL . Magnetic resonance‐compatible arm‐crank ergometry: A new platform linking whole‐body calorimetry to upper‐extremity biomechanics and arm muscle metabolism. Front Physiol 2021;12:599514. 33679429 10.3389/fphys.2021.599514 PMC7933461

[27] Habets LE , Bartels B , Asselman F‐L , et al. Magnetic resonance reveals mitochondrial dysfunction and muscle remodelling in spinal muscular atrophy. Brain 2022;145:1422‐1435. 34788410 10.1093/brain/awab411 PMC9128825

[28] Wong DTH , Lee K‐J , Yoo S‐J , Tomlinson G , Grosse‐Wortmann L . Changes in systemic and pulmonary blood flow distribution in normal adult volunteers in response to posture and exercise: A phase contrast magnetic resonance imaging study. J Physiol Sci 2014;64:105‐112. 24385190 10.1007/s12576-013-0298-z PMC10717753

[29] Dillon HT , Dausin C , Claessen G , et al. The effect of posture on maximal oxygen uptake in active healthy individuals. Eur J Appl Physiol 2021;121:1487‐1498. 33638017 10.1007/s00421-021-04630-7

[30] Prompers JJ , Jeneson JAL , Drost MR , Oomens CCW , Strijkers GJ , Nicolay K . Dynamic MRS and MRI of skeletal muscle function and biomechanics. NMR Biomed 2006;19:927‐953. 17075956 10.1002/nbm.1095

[31] Meyerspeer M , Boesch C , Cameron D , et al. ^31^P magnetic resonance spectroscopy in skeletal muscle: Experts' consensus recommendations. NMR Biomed 2020;34:e4246. 32037688 10.1002/nbm.4246 PMC8243949

[32] Sedivy P , Dezortova M , Rydlo J , et al. MR compatible ergometers for dynamic ^31^P MRS. J Appl Biomed 2019;17:91‐98. 34907736 10.32725/jab.2019.006

[33] Valkovič L , Chmelík M , Ukropcová B , et al. Skeletal muscle alkaline P_i_ pool is decreased in overweight‐to‐obese sedentary subjects and relates to mitochondrial capacity and phosphodiester content. Sci Rep 2016;6:20087. 26838588 10.1038/srep20087 PMC4738275

[34] Wijma AG , Driessens H , Jeneson JAL , et al. Cardiac and intramuscular adaptations following short‐term exercise prehabilitation in unfit patients scheduled to undergo hepatic or pancreatic surgery: Study protocol of a multinuclear MRI study. BMJ Open Gastroenterol 2023;10:e001243. 10.1136/bmjgast-2023-001243 PMC10668156 37996121

[35] Lanza IR , Bhagra S , Nair KS , Port JD . Measurement of human skeletal muscle oxidative capacity by ^31^P‐MR spectroscopy: A cross‐validation with *in vitro* measurements. J Magn Reson Imaging 2011;34:1143‐1150. 22006551 10.1002/jmri.22733 PMC3201762

[36] Kemp GJ , Hands LJ , Ramaswami G , et al. Calf muscle mitochondrial and glycogenolytic ATP synthesis in patients with claudication due to peripheral vascular disease analysed using ^31^P magnetic resonance spectroscopy. Clin Sci 1995;89:581‐590. 10.1042/cs0890581 8549076

[37] Taylor DJ , Amato A , Hands LJ , et al. Changes in energy metabolism of calf muscle in patients with intermittent claudication assessed by ^31^P magnetic resonance spectroscopy: A phase II open study. Vasc Med 1996;1:241‐245. 9552578 10.1177/1358863X9600100401

[38] Cea G , Bendahan D , Manners D , et al. Reduced oxidative phosphorylation and proton efflux suggest reduced capillary blood supply in skeletal muscle of patients with dermatomyositis and polymyositis: A quantitative ^31^P‐magnetic resonance spectroscopy and MRI study. Brain 2002;125(Pt 7):1635‐1645. 12077012 10.1093/brain/awf163

[39] Taylor DJ . Clinical utility of muscle MR spectroscopy. Semin Musculoskelet Radiol 2000;4:481‐502. 11371330 10.1055/s-2000-13172

[40] Park JH , Brown RL , Park CR , Cohn M , Chance B . Energy metabolism of the untrained muscle of elite runners as observed by ^31^P magnetic resonance spectroscopy: Evidence suggesting a genetic endowment for endurance exercise. Proc Natl Acad Sci U S A 1988;85:8780‐8784. 3194388 10.1073/pnas.85.23.8780 PMC282590

[41] Yoshida T , Watari H . Exercise‐induced splitting of the inorganic phosphate peak: Investigation by time‐resolved ^31^P‐nuclear magnetic resonance spectroscopy. Eur J Appl Physiol Occup Physiol 1994;69:465‐473. 7713064 10.1007/BF00239861

[42] Mizuno M , Horn A , Secher NH , Quistorff B . Exercise‐induced ^31^P‐NMR metabolic response of human wrist flexor muscles during partial neuromuscular blockade. Am J Physiol 1994;267(2 Pt 2):R408‐R414. 7915086 10.1152/ajpregu.1994.267.2.R408

[43] Hooijmans MT , Niks EH , Burakiewicz J , et al. Non‐uniform muscle fat replacement along the proximodistal axis in Duchenne muscular dystrophy. Neuromuscul Disord 2017;27:458‐464. 28302391 10.1016/j.nmd.2017.02.009

[44] Buchthal SD , Thoma WJ , Taylor JS , Nelson SJ , Brown TR . *In vivo* T_1_ values of phosphorus metabolites in human liver and muscle determined at 1.5 T by chemical shift imaging. NMR Biomed 1989;2:298‐304. 2641903 10.1002/nbm.1940020520

[45] Slade JM , Towse TF , DeLano MC , Wiseman RW , Meyer RA . A gated ^31^P NMR method for the estimation of phosphocreatine recovery time and contractile ATP cost in human muscle. NMR Biomed 2006;19:573‐580. 16642462 10.1002/nbm.1037

[46] Boss A , Heskamp L , Breukels V , Bains LJ , van Uden MJ , Heerschap A . Oxidative capacity varies along the length of healthy human tibialis anterior. J Physiol 2018;596:1467‐1483. 29455454 10.1113/JP275009 PMC5899983

[47] Valkovič L , Chmelík M , Just Kukurová I , et al. Depth‐resolved surface coil MRS (DRESS)‐localized dynamic ^31^P‐MRS of the exercising human gastrocnemius muscle at 7 T. NMR Biomed 2014;27:1346‐1352. 25199902 10.1002/nbm.3196

[48] Forbes SC , Slade JM , Francis RM , Meyer RA . Comparison of oxidative capacity among leg muscles in humans using gated ^31^P 2‐D chemical shift imaging. NMR Biomed 2009;22:1063‐1071. 19579230 10.1002/nbm.1413

[49] Parasoglou P , Xia D , Regatte RR . Spectrally selective 3D TSE imaging of phosphocreatine in the human calf muscle at 3 T. Magn Reson Med 2013;69:812‐817. 22499078 10.1002/mrm.24288 PMC3402708

[50] Schmid AI , Meyerspeer M , Robinson SD , et al. Dynamic PCr and pH imaging of human calf muscles during exercise and recovery using ^31^P gradient‐Echo MRI at 7 Tesla. Magn Reson Med 2016;75:2324‐2331. 26115021 10.1002/mrm.25822

[51] Krumpolec P , Klepochová R , Just I , et al. Multinuclear MRS at 7T uncovers exercise driven differences in skeletal muscle energy metabolism between young and seniors. Front Physiol 2020;11:644. 32695010 10.3389/fphys.2020.00644 PMC7336536

[52] Menon RG , Xia D , Katz SD , Regatte RR . Dynamic ^31^P‐MRI and ^31^P‐MRS of lower leg muscles in heart failure patients. Sci Rep 2021;11:7412. 33795721 10.1038/s41598-021-86392-y PMC8016929

[53] Befroy DE , Falk Petersen K , Rothman DL , Shulman GI . Assessment of *in vivo* mitochondrial metabolism by magnetic resonance spectroscopy. Methods Enzymol 2009;457:373‐393. 19426879 10.1016/S0076-6879(09)05021-6 PMC3077057

[54] Kogan F , Haris M , Singh A , et al. Method for high‐resolution imaging of creatine *in vivo* using chemical exchange saturation transfer. Magn Reson Med 2014;71:164‐172. 23412909 10.1002/mrm.24641 PMC3725192

[55] Kemp GJ , Brindle KM . What do magnetic resonance‐based measurements of P_i_→ATP flux tell us about skeletal muscle metabolism? Diabetes 2012;61:1927‐1934. 22826313 10.2337/db11-1725 PMC3402329

[56] Sleigh A , Savage DB , Williams GB , et al. ^31^P magnetization transfer measurements of P_i_→ATP flux in exercising human muscle. J Appl Physiol 1985;2016(120):649‐656. 10.1152/japplphysiol.00871.2015 PMC4796179 26744504

[57] DeBrosse C , Nanga RPR , Wilson N , et al. Muscle oxidative phosphorylation quantitation using creatine chemical exchange saturation transfer (CrCEST) MRI in mitochondrial disorders. JCI Insight 2016;1:e88207. 27812541 10.1172/jci.insight.88207 PMC5085612

[58] Schur GM , Dunn J , Nguyen S , et al. *In vivo* assessment of OXPHOS capacity using 3 T CrCEST MRI in Friedreich's ataxia. J Neurol 2022;269:2527‐2538. 34652504 10.1007/s00415-021-10821-1 PMC9010488

[59] DeLorey DS , Clifford PS . Does sympathetic vasoconstriction contribute to metabolism: Perfusion matching in exercising skeletal muscle? Front Physiol 2022;13:980524. 36171966 10.3389/fphys.2022.980524 PMC9510655

[60] Towse TF , Slade JM , Ambrose JA , DeLano MC , Meyer RA . Quantitative analysis of the postcontractile blood‐oxygenation‐level‐dependent (BOLD) effect in skeletal muscle. J Appl Physiol 1985;2011(111):27‐39. 10.1152/japplphysiol.01054.2009 PMC3137544 21330621

[61] Partovi S , Aschwanden M , Jacobi B , et al. Correlation of muscle BOLD MRI with transcutaneous oxygen pressure for assessing microcirculation in patients with systemic sclerosis. J Magn Reson Imaging 2013;38:845‐851. 23441019 10.1002/jmri.24046

[62] Partovi S , Schulte A‐C , Staub D , et al. Correlation of skeletal muscle blood oxygenation level‐dependent MRI and skin laser Doppler flowmetry in patients with systemic sclerosis. J Magn Reson Imaging 2014;40:1408‐1413. 24338875 10.1002/jmri.24503

[63] Elder CP , Cook RN , Chance MA , Copenhaver EA , Damon BM . Image‐based calculation of perfusion and oxyhemoglobin saturation in skeletal muscle during submaximal isometric contractions. Magn Reson Med 2010;64:852‐861. 20806379 10.1002/mrm.22475 PMC4437700

[64] Sanchez OA , Copenhaver EA , Elder CP , Damon BM . Absence of a significant extravascular contribution to the skeletal muscle BOLD effect at 3 T. Magn Reson Med 2010;64:527‐535. 20665796 10.1002/mrm.22449 PMC2914541

[65] Pollak AW , Meyer CH , Epstein FH , et al. Arterial spin labeling MR imaging reproducibly measures peak‐exercise calf muscle perfusion: A study in patients with peripheral arterial disease and healthy volunteers. JACC Cardiovasc Imaging 2012;5:1224‐1230. 23236972 10.1016/j.jcmg.2012.03.022 PMC3531823

[66] Sanchez OA , Copenhaver EA , Chance MA , et al. Postmaximal contraction blood volume responses are blunted in obese and type 2 diabetic subjects in a muscle‐specific manner. Am J Physiol Heart Circ Physiol 2011;301:H418‐H427. 21572006 10.1152/ajpheart.00060.2011 PMC3154659

[67] Conlin CC , Layec G , Hanrahan CJ , et al. Exercise‐stimulated arterial transit time in calf muscles measured by dynamic contrast‐enhanced magnetic resonance imaging. Physiol Rep 2019;7:e13978. 30648355 10.14814/phy2.13978 PMC6333626

[68] Detre JA , Leigh JS , Williams DS , Koretsky AP . Perfusion imaging. Magn Reson Med 1992;23:37‐45. 1734182 10.1002/mrm.1910230106

[69] Veeger TTJ , Hirschler L , Baligand C , et al. Microvascular response to exercise varies along the length of the tibialis anterior muscle. NMR Biomed 2022;35:e4796. 35778859 10.1002/nbm.4796 PMC9787660

[70] Le Bihan D . What can we see with IVIM MRI? Neuroimage 2019;187:56‐67. 29277647 10.1016/j.neuroimage.2017.12.062

[71] Adelnia F , Shardell M , Bergeron CM , et al. Diffusion‐weighted MRI with intravoxel incoherent motion modeling for assessment of muscle perfusion in the thigh during post‐exercise hyperemia in younger and older adults. NMR Biomed 2019;32:e4072. 30861224 10.1002/nbm.4072 PMC6530599

[72] Wray DW , Nishiyama SK , Monnet A , et al. Multiparametric NMR‐based assessment of skeletal muscle perfusion and metabolism during exercise in elderly persons: Preliminary findings. J Gerontol A Biol Sci Med Sci 2009;64:968‐974. 19377015 10.1093/gerona/glp044 PMC2720884

[73] Schulte A‐C , Aschwanden M , Bilecen D . Calf muscles at blood oxygen level‐dependent MR imaging: Aging effects at postocclusive reactive hyperemia. Radiology 2008;247:482‐489. 18372453 10.1148/radiol.2472070828

[74] Wang ZY , Noyszewski EA , Leigh JS . *In vivo* MRS measurement of deoxymyoglobin in human forearms. Magn Reson Med 1990;14:562‐567. 2355838 10.1002/mrm.1910140314

[75] Richardson RS , Noyszewski EA , Kendrick KF , Leigh JS , Wagner PD . Myoglobin O_2_ desaturation during exercise. Evidence of limited O_2_ transport. J Clin Invest 1995;96:1916‐1926. 7560083 10.1172/JCI118237 PMC185828

[76] Kreis R , Bruegger K , Skjelsvik C , et al. Quantitative ^1^H magnetic resonance spectroscopy of myoglobin de‐ and reoxygenation in skeletal muscle: Reproducibility and effects of location and disease. Magn Reson Med 2001;46:240‐248. 11477626 10.1002/mrm.1184

[77] Wary C , Nadaj‐Pakleza A , Laforêt P , et al. Investigating glycogenosis type III patients with multi‐parametric functional NMR imaging and spectroscopy. Neuromuscul Disord 2010;20:548‐558. 20620060 10.1016/j.nmd.2010.06.011

[78] Quaresima V , Farzam P , Anderson P , et al. Diffuse correlation spectroscopy and frequency‐domain near‐infrared spectroscopy for measuring microvascular blood flow in dynamically exercising human muscles. J Appl Physiol 1985;2019(127):1328‐1337. 10.1152/japplphysiol.00324.2019 31513443

[79] Bartlett MF , Oneglia AP , Ricard MD , et al. DCS blood flow index underestimates skeletal muscle perfusion *in vivo*: Rationale and early evidence for the NIRS‐DCS perfusion index. J Biomed Opt 2024;29:020501. 38322728 10.1117/1.JBO.29.2.020501 PMC10844820

[80] Neprokin A , Broadway C , Myllylä T , Bykov A , Meglinski I . Photoacoustic imaging in biomedicine and life sciences. Life 2022;12:588. 35455079 10.3390/life12040588 PMC9028050

[81] Lopez Kolkovsky AL , Marty B , Giacomini E , Meyerspeer M , Carlier PG . Repeatability of multinuclear interleaved acquisitions with nuclear Overhauser enhancement effect in dynamic experiments in the calf muscle at 3T. Magn Reson Med 2021;86:115‐130. 33565187 10.1002/mrm.28684

[82] Suo S , Zhang L , Tang H , et al. Evaluation of skeletal muscle microvascular perfusion of lower extremities by cardiovascular magnetic resonance arterial spin labeling, blood oxygenation level‐dependent, and intravoxel incoherent motion techniques. J Cardiovasc Magn Reson 2018;20:18. 29551091 10.1186/s12968-018-0441-3 PMC5858129

[83] Caroca S , Villagran D , Chabert S . Four functional magnetic resonance imaging techniques for skeletal muscle exploration, a systematic review. Eur J Radiol 2021;144:109995. 34628310 10.1016/j.ejrad.2021.109995

[84] Ohno N , Miyati T , Fujihara S , Gabata T , Kobayashi S . Biexponential analysis of intravoxel incoherent motion in calf muscle before and after exercise: Comparisons with arterial spin labeling perfusion and T_2_ . Magn Reson Imaging 2020;72:42‐48. 32561379 10.1016/j.mri.2020.06.003

[85] Sleutjes BTHM , Drenthen J , Boskovic E , et al. Excitability tests using high‐density surface‐EMG: A novel approach to studying single motor units. Clin Neurophysiol 2018;129:1634‐1641. 29909363 10.1016/j.clinph.2018.04.754

[86] Csapo R , Malis V , Sinha U , Sinha S . Mapping of spatial and temporal heterogeneity of plantar flexor muscle activity during isometric contraction: Correlation of velocity‐encoded MRI with EMG. J Appl Physiol 1985;2015(119):558‐568. 10.1152/japplphysiol.00275.2015 PMC4556836 26112239

[87] Hooijmans MT , Schlaffke L , Bolsterlee B , Schlaeger S , Marty B , Mazzoli V . Compositional and functional MRI of skeletal muscle: A review. J Magn Reson Imaging 2024;60(3):860‐877. 10.1002/jmri.29091. 37929681 PMC11070452

[88] Pappas GP , Asakawa DS , Delp SL , Zajac FE , Drace JE . Nonuniform shortening in the biceps brachii during elbow flexion. J Appl Physiol 1985;2002(92):2381‐2389. 10.1152/japplphysiol.00843.2001 12015351

[89] Finni T , Hodgson JA , Lai AM , Edgerton VR , Sinha S . Mapping of movement in the isometrically contracting human soleus muscle reveals details of its structural and functional complexity. J Appl Physiol 1985;2003(95):2128‐2133. 10.1152/japplphysiol.00596.2003 12857769

[90] Csapo R , Malis V , Hodgson J , Sinha S . Age‐related greater Achilles tendon compliance is not associated with larger plantar flexor muscle fascicle strains in senior women. J Appl Physiol 1985;2014(116):961‐969. 10.1152/japplphysiol.01337.2013 PMC4035789 24505104

[91] Silder A , Reeder SB , Thelen DG . The influence of prior hamstring injury on lengthening muscle tissue mechanics. J Biomech 2010;43:2254‐2260. 20472238 10.1016/j.jbiomech.2010.02.038 PMC2922492

[92] Sinha U , Malis V , Csapo R , Moghadasi A , Kinugasa R , Sinha S . Age‐related differences in strain rate tensor of the medial gastrocnemius muscle during passive plantarflexion and active isometric contraction using velocity encoded MR imaging: Potential index of lateral force transmission. Magn Reson Med 2015;73:1852‐1863. 25046255 10.1002/mrm.25312 PMC4287463

[93] Malis V , Sinha U , Csapo R , Narici M , Sinha S . Relationship of changes in strain rate indices estimated from velocity‐encoded MR imaging to loss of muscle force following disuse atrophy. Magn Reson Med 2018;79:912‐922. 28560822 10.1002/mrm.26759 PMC5709278

[94] Schlaffke L , Rehmann R , Rohm M , et al. Multi‐center evaluation of stability and reproducibility of quantitative MRI measures in healthy calf muscles. NMR Biomed 2019;32:e4119. 31313867 10.1002/nbm.4119

[95] Heskamp L , Lebbink F , van Uden MJ , et al. Post‐exercise intramuscular O_2_ supply is tightly coupled with a higher proximal‐to‐distal ATP synthesis rate in human tibialis anterior. J Physiol 2021;599:1533‐1550. 33369737 10.1113/JP280771 PMC7986184

[96] Mazzoli V , Gottwald LM , Peper ES , et al. Accelerated 4D phase contrast MRI in skeletal muscle contraction. Magn Reson Med 2018;80:1799‐1811. 29508449 10.1002/mrm.27158

[97] Malis V , Sinha U , Sinha S . Compressed sensing velocity encoded phase contrast imaging: Monitoring skeletal muscle kinematics. Magn Reson Med 2020;84:142‐156. 31828833 10.1002/mrm.28100 PMC8046431

[98] Hooijmans MT , Veeger TTJ , Mazzoli V , et al. Muscle fiber strain rates in the lower leg during ankle dorsi‐/plantarflexion exercise. NMR Biomed 2024;37:e5064. 38062865 10.1002/nbm.5064

[99] Mazzoli V , Moulin K , Kogan F , Hargreaves BA , Gold GE . Diffusion tensor imaging of skeletal muscle contraction using oscillating gradient spin echo. Front Neurol 2021;12:608549. 33658976 10.3389/fneur.2021.608549 PMC7917051

[100] Steidle G , Schick F . Addressing spontaneous signal voids in repetitive single‐shot DWI of musculature: Spatial and temporal patterns in the calves of healthy volunteers and consideration of unintended muscle activities as underlying mechanism. NMR Biomed 2015;28:801‐810. 25943431 10.1002/nbm.3311

[101] Heskamp L , Birkbeck MG , Baxter‐Beard D , et al. Motor unit magnetic resonance imaging (MUMRI) in skeletal muscle. J Magn Reson Imaging 2024;60(6):2253‐2271. 10.1002/jmri.29218. 38216545

[102] Navarro‐Valverde C , Ramos‐Maqueda J , Romero‐Reyes MJ , et al. Magnetic resonance imaging in patients with cardiac implantable electronic devices: A prospective study. Magn Reson Imaging 2022;91:9‐15. 35526803 10.1016/j.mri.2022.05.004

[103] Xiang J , Lamy J , Lampert R , Peters DC . Balanced steady‐state free precession cine MR imaging in the presence of cardiac devices: Value of interleaved radial linear combination acquisition with partial dephasing. J Magn Reson Imaging 2023;58:782‐791. 36373998 10.1002/jmri.28528 PMC11238270

[104] Niezen RA , Doornbos J , van der Wall EE , de Roos A . Measurement of aortic and pulmonary flow with MRI at rest and during physical exercise. J Comput Assist Tomogr 1998;22:194‐201. 9530378 10.1097/00004728-199803000-00006

[105] Mayr A , Klug G , Reinstadler SJ , et al. Cardiac exercise imaging using a 3‐tesla magnetic resonance‐conditional pedal ergometer: Preliminary results in healthy volunteers and patients with known or suspected coronary artery disease. Cardiol J 2023;30:276‐285. 34490601 10.5603/CJ.a2021.0095 PMC10129251

[106] Kramer CM , Barkhausen J , Bucciarelli‐Ducci C , Flamm SD , Kim RJ , Nagel E . Standardized cardiovascular magnetic resonance imaging (CMR) protocols: 2020 update. J Cardiovasc Magn Reson 2020;22:17. 32089132 10.1186/s12968-020-00607-1 PMC7038611

[107] Craven TP , Tsao CW , La Gerche A , Simonetti OP , Greenwood JP . Exercise cardiovascular magnetic resonance: Development, current utility and future applications. J Cardiovasc Magn Reson 2020;22:65. 32907587 10.1186/s12968-020-00652-w PMC7488086

[108] Mohiaddin RH , Gatehouse PD , Firmin DN . Exercise‐related changes in aortic flow measured with spiral echo‐planar MR velocity mapping. J Magn Reson Imaging 1995;5:159‐163. 7766976 10.1002/jmri.1880050209

[109] Pedersen EM , Kozerke S , Ringgaard S , Scheidegger MB , Boesiger P . Quantitative abdominal aortic flow measurements at controlled levels of ergometer exercise. Magn Reson Imaging 1999;17:489‐494. 10231175 10.1016/s0730-725x(98)00209-4

[110] Darr KC , Bassett DR , Morgan BJ , Thomas DP . Effects of age and training status on heart rate recovery after peak exercise. Am J Physiol 1988;254(2 Pt 2):H340‐H343. 3344824 10.1152/ajpheart.1988.254.2.H340

[111] Roberts PA , Cowan BR , Liu Y , et al. Real‐time aortic pulse wave velocity measurement during exercise stress testing. J Cardiovasc Magn Reson 2015;17:86. 26438096 10.1186/s12968-015-0191-4 PMC4594994

[112] Forouzan O , Warczytowa J , Wieben O , François CJ , Chesler NC . Non‐invasive measurement using cardiovascular magnetic resonance of changes in pulmonary artery stiffness with exercise. J Cardiovasc Magn Reson 2015;17:109. 26653289 10.1186/s12968-015-0213-2 PMC4677443

[113] Stevens GR , Garcia‐Alvarez A , Sahni S , Garcia MJ , Fuster V , Sanz J . RV dysfunction in pulmonary hypertension is independently related to pulmonary artery stiffness. JACC Cardiovasc Imaging 2012;5:378‐387. 22498327 10.1016/j.jcmg.2011.11.020

[114] Macdonald JA , Beshish AG , Corrado PA , et al. Feasibility of cardiovascular four‐dimensional flow MRI during exercise in healthy participants. Radiol Cardiothorac Imaging 2020;2:e190033. 32734274 10.1148/ryct.2020190033 PMC7373355

[115] Macdonald JA , Roberts GS , Corrado PA , et al. Exercise‐induced irregular right heart flow dynamics in adolescents and young adults born preterm. J Cardiovasc Magn Reson 2021;23:116. 34670573 10.1186/s12968-021-00816-2 PMC8529801

[116] Pedersen EM , Stenbøg EV , Fründ T , et al. Flow during exercise in the total cavopulmonary connection measured by magnetic resonance velocity mapping. Heart 2002;87:554‐558. 12010939 10.1136/heart.87.6.554 PMC1767137

[117] Hjortdal VE , Emmertsen K , Stenbøg E , et al. Effects of exercise and respiration on blood flow in total cavopulmonary connection: A real‐time magnetic resonance flow study. Circulation 2003;108:1227‐1231. 12939218 10.1161/01.CIR.0000087406.27922.6B

[118] Weber TF , von Tengg‐Kobligk H , Kopp‐Schneider A , Ley‐Zaporozhan J , Kauczor H‐U , Ley S . High‐resolution phase‐contrast MRI of aortic and pulmonary blood flow during rest and physical exercise using a MRI compatible bicycle ergometer. Eur J Radiol 2011;80:103‐108. 20674204 10.1016/j.ejrad.2010.06.045

[119] Holverda S , Gan CT‐J , Marcus JT , Postmus PE , Boonstra A , Vonk‐Noordegraaf A . Impaired stroke volume response to exercise in pulmonary arterial hypertension. J Am Coll Cardiol 2006;47:1732‐1733. 16631018 10.1016/j.jacc.2006.01.048

[120] Schulz‐Menger J , Bluemke DA , Bremerich J , et al. Standardized image interpretation and post‐processing in cardiovascular magnetic resonance – 2020 update. J Cardiovasc Magn Reson 2020;22:19. 32160925 10.1186/s12968-020-00610-6 PMC7066763

[121] Heiberg J , Asschenfeldt B , Maagaard M , Ringgaard S . Dynamic bicycle exercise to assess cardiac output at multiple exercise levels during magnetic resonance imaging. Clin Imaging 2017;46:102‐107. 28778011 10.1016/j.clinimag.2017.07.010

[122] Macdonald JA , Franҫois CJ , Forouzan O , Chesler NC , Wieben O . MRI assessment of aortic flow in patients with pulmonary arterial hypertension in response to exercise. BMC Med Imaging 2018;18:55. 30577768 10.1186/s12880-018-0298-9 PMC6303959

[123] Edlund J , Haris K , Ostenfeld E , et al. Validation and quantification of left ventricular function during exercise and free breathing from real‐time cardiac magnetic resonance images. Sci Rep 2022;12:5611. 35379859 10.1038/s41598-022-09366-8 PMC8979972

[124] Uretsky S , Argulian E , Narula J , Wolff SD . Use of cardiac magnetic resonance imaging in assessing mitral regurgitation: Current evidence. J Am Coll Cardiol 2018;71:547‐563. 29406861 10.1016/j.jacc.2017.12.009

[125] Roest AA , Kunz P , Lamb HJ , Helbing WA , van der Wall EE , de Roos A . Biventricular response to supine physical exercise in young adults assessed with ultrafast magnetic resonance imaging. Am J Cardiol 2001;87:601‐605. 11230846 10.1016/s0002-9149(00)01438-7

[126] Beaudry RI , Samuel TJ , Wang J , Tucker WJ , Haykowsky MJ , Nelson MD . Exercise cardiac magnetic resonance imaging: A feasibility study and meta‐analysis. Am J Physiol Regul Integr Comp Physiol 2018;315:R638‐R645. 29949409 10.1152/ajpregu.00158.2018 PMC6230887

[127] Nayak KS , Lim Y , Campbell‐Washburn AE , Steeden J . Real‐time magnetic resonance imaging. J Magn Reson Imaging 2022;55:81‐99. 33295674 10.1002/jmri.27411 PMC8435094

[128] Lurz P , Muthurangu V , Schievano S , et al. Feasibility and reproducibility of biventricular volumetric assessment of cardiac function during exercise using real‐time radial *k*‐*t* SENSE magnetic resonance imaging. J Magn Reson Imaging 2009;29:1062‐1070. 19388126 10.1002/jmri.21762

[129] Steinmetz M , Stümpfig T , Seehase M , et al. Impaired exercise tolerance in repaired tetralogy of Fallot is associated with impaired biventricular contractile reserve: An exercise‐stress real‐time cardiovascular magnetic resonance study. Circ Cardiovasc Imaging 2021;14:e011823. 34384226 10.1161/CIRCIMAGING.120.011823

[130] La Gerche A , Claessen G , Van de Bruaene A , et al. Cardiac MRI: A new gold standard for ventricular volume quantification during high‐intensity exercise. Circ Cardiovasc Imaging 2013;6:329‐338. 23258478 10.1161/CIRCIMAGING.112.980037

[131] Le T‐T , Bryant JA , Ting AE , et al. Assessing exercise cardiac reserve using real‐time cardiovascular magnetic resonance. J Cardiovasc Magn Reson 2017;19:7. 28110638 10.1186/s12968-017-0322-1 PMC5256575

[132] Kirkham AA , Goonasekera MV , Mattiello BC , Grenier JG , Haykowsky MJ , Thompson RB . Reliability and reproducibility of cardiac MRI quantification of peak exercise function with long‐axis views. PLoS One 2021;16:e0245912. 33539447 10.1371/journal.pone.0245912 PMC7861545

[133] Novillo F , Van Eyndhoven S , Moeyersons J , et al. Unsupervised respiratory signal extraction from ungated cardiac magnetic resonance imaging at rest and during exercise. Phys Med Biol 2019;64:065001. 30695762 10.1088/1361-6560/ab02cd

[134] Chen C , Chandrasekaran P , Liu Y , Simonetti OP , Tong M , Ahmad R . Ensuring respiratory phase consistency to improve cardiac function quantification in real‐time CMR. Magn Reson Med 2022;87:1595‐1604. 34719067 10.1002/mrm.29064 PMC8776600

[135] Backhaus SJ , Metschies G , Billing M , et al. Defining the optimal temporal and spatial resolution for cardiovascular magnetic resonance imaging feature tracking. J Cardiovasc Magn Reson 2021;23:60. 34001175 10.1186/s12968-021-00740-5 PMC8127257

[136] Li YY , Zhang P , Rashid S , et al. Real‐time exercise stress cardiac MRI with Fourier‐series reconstruction from golden‐angle radial data. Magn Reson Imaging 2021;75:89‐99. 33098934 10.1016/j.mri.2020.10.010 PMC7683360

[137] Uecker M , Zhang S , Voit D , Karaus A , Merboldt K‐D , Frahm J . Real‐time MRI at a resolution of 20 ms. NMR Biomed 2010;23:986‐994. 20799371 10.1002/nbm.1585

[138] La Gerche A , Claessen G , Dymarkowski S , et al. Exercise‐induced right ventricular dysfunction is associated with ventricular arrhythmias in endurance athletes. Eur Heart J 2015;36:1998‐2010. 26038590 10.1093/eurheartj/ehv202

[139] Foulkes SJ , Howden EJ , Haykowsky MJ , et al. Exercise for the prevention of anthracycline‐induced functional disability and cardiac dysfunction: The BREXIT study. Circulation 2023;147:532‐545. 36342348 10.1161/CIRCULATIONAHA.122.062814

[140] Latus H , Hofmann L , Gummel K , et al. Exercise‐dependent changes in ventricular‐arterial coupling and aortopulmonary collateral flow in Fontan patients: A real‐time CMR study. Eur Heart J Cardiovasc Imaging 2022;24:88‐97. 35045176 10.1093/ehjci/jeac001 PMC9762934

[141] Yang W , Xu J , Zhu L , et al. Myocardial strain measurements derived from MR feature‐tracking: Influence of sex, age, field strength, and vendor. JACC Cardiovasc Imaging 2024;17:364‐379. 37480906 10.1016/j.jcmg.2023.05.019

[142] Lin ACW , Seale H , Hamilton‐Craig C , Morris NR , Strugnell W . Quantification of biventricular strain and assessment of ventriculo‐ventricular interaction in pulmonary arterial hypertension using exercise cardiac magnetic resonance imaging and myocardial feature tracking. J Magn Reson Imaging 2019;49:1427‐1436. 30353959 10.1002/jmri.26517

[143] Backhaus SJ , Lange T , George EF , et al. Exercise stress real‐time cardiac magnetic resonance imaging for noninvasive characterization of heart failure with preserved ejection fraction: The HFpEF‐Stress trial. Circulation 2021;143:1484‐1498. 33472397 10.1161/CIRCULATIONAHA.120.051542

[144] Sampath S , Derbyshire JA , Ledesma‐Carbayo MJ , McVeigh ER . Imaging left ventricular tissue mechanics and hemodynamics during supine bicycle exercise using a combined tagging and phase‐contrast MRI pulse sequence. Magn Reson Med 2011;65:51‐59. 21053325 10.1002/mrm.22668

[145] Mohammadi E , Nasiraei‐Moghaddam A , Uecker M . Real‐time radial tagging for quantification of left ventricular torsion. Magn Reson Med 2022;87:2741‐2756. 35081262 10.1002/mrm.29169

[146] Morales MA , Yoon S , Fahmy A , et al. Highly accelerated free‐breathing real‐time myocardial tagging for exercise cardiovascular magnetic resonance. J Cardiovasc Magn Reson 2023;25:56. 37784153 10.1186/s12968-023-00961-w PMC10544487

[147] Knuuti J , Wijns W , Saraste A , et al. 2019 ESC Guidelines for the diagnosis and management of chronic coronary syndromes. Eur Heart J 2020;41:407‐477. 31504439 10.1093/eurheartj/ehz425

[148] Patel AR , Salerno M , Kwong RY , Singh A , Heydari B , Kramer CM . Stress cardiac magnetic resonance myocardial perfusion imaging: JACC review topic of the week. J Am Coll Cardiol 2021;78:1655‐1668. 34649703 10.1016/j.jacc.2021.08.022 PMC8530410

[149] Basha TA , Roujol S , Kissinger KV , et al. Free‐breathing cardiac MR stress perfusion with real‐time slice tracking. Magn Reson Med 2014;72:689‐698. 24123153 10.1002/mrm.24977 PMC3979504

[150] Pflugi S , Roujol S , Akçakaya M , et al. Accelerated cardiac MR stress perfusion with radial sampling after physical exercise with an MR‐compatible supine bicycle ergometer. Magn Reson Med 2015;74:384‐395. 25105469 10.1002/mrm.25405

[151] Le T‐T , Ang BWY , Bryant JA , et al. Multiparametric exercise stress cardiovascular magnetic resonance in the diagnosis of coronary artery disease: The EMPIRE trial. J Cardiovasc Magn Reson 2021;23:17. 33658056 10.1186/s12968-021-00705-8 PMC7931509

[152] Nickander J , Themudo R , Thalén S , et al. The relative contributions of myocardial perfusion, blood volume and extracellular volume to native T_1_ and native T_2_ at rest and during adenosine stress in normal physiology. J Cardiovasc Magn Reson 2019;21:73. 31767018 10.1186/s12968-019-0585-9 PMC6876099

[153] Liu A , Wijesurendra RS , Francis JM , et al. Adenosine stress and rest T_1_ mapping can differentiate between ischemic, infarcted, remote, and normal myocardium without the need for gadolinium contrast agents. JACC Cardiovasc Imaging 2016;9:27‐36. 26684978 10.1016/j.jcmg.2015.08.018 PMC4708879

[154] Nakamori S , Fahmy A , Jang J , et al. Changes in myocardial native T_1_ and T_2_ after exercise stress: A noncontrast CMR pilot study. JACC Cardiovasc Imaging 2020;13:667‐680. 31326484 10.1016/j.jcmg.2019.05.019

[155] Gobel FL , Norstrom LA , Nelson RR , Jorgensen CR , Wang Y . The rate‐pressure product as an index of myocardial oxygen consumption during exercise in patients with angina pectoris. Circulation 1978;57:549‐556. 624164 10.1161/01.cir.57.3.549

[156] Guo R , Qi H , Amyar A , et al. Quantification of changes in myocardial T_1_* values with exercise cardiac MRI using a free‐breathing non‐electrocardiograph radial imaging. Magn Reson Med 2022;88:1720‐1733. 35691942 10.1002/mrm.29346

[157] Globits S , Sakuma H , Shimakawa A , Foo TK , Higgins CB . Measurement of coronary blood flow velocity during handgrip exercise using breath‐hold velocity encoded cine magnetic resonance imaging. Am J Cardiol 1997;79:234‐237. 9193037 10.1016/s0002-9149(97)89291-0

[158] Hays AG , Hirsch GA , Kelle S , Gerstenblith G , Weiss RG , Stuber M . Noninvasive visualization of coronary artery endothelial function in healthy subjects and in patients with coronary artery disease. J Am Coll Cardiol 2010;56:1657‐1665. 21050976 10.1016/j.jacc.2010.06.036

[159] Bottomley PA . Noninvasive study of high‐energy phosphate metabolism in human heart by depth‐resolved ^31^P NMR spectroscopy. Science 1985;229:769‐772. 4023711 10.1126/science.4023711

[160] Conway MA , Bristow JD , Blackledge MJ , Rajagopalan B , Radda GK . Cardiac metabolism during exercise measured by magnetic resonance spectroscopy. Lancet 1988;2:692. 2901555 10.1016/s0140-6736(88)90510-7

[161] Weiss RG , Bottomley PA , Hardy CJ , Gerstenblith G . Regional myocardial metabolism of high‐energy phosphates during isometric exercise in patients with coronary artery disease. N Engl J Med 1990;323:1593‐1600. 2233948 10.1056/NEJM199012063232304

[162] Conway MA , Bristow JD , Blackledge MJ , Rajagopalan B , Radda GK . Cardiac metabolism during exercise in healthy volunteers measured by ^31^P magnetic resonance spectroscopy. Br Heart J 1991;65:25‐30. 1993127 10.1136/hrt.65.1.25 PMC1024458

[163] Kuno S , Ogawa T , Katsuta S , Itai Y . *In vivo* human myocardial metabolism during aerobic exercise by phosphorus‐31 nuclear magnetic resonance spectroscopy. Eur J Appl Physiol Occup Physiol 1994;69:488‐491. 7713067 10.1007/BF00239864

[164] Ordidge RJ , Connelly A , Lohman JAB . Image‐selected *in vivo* spectroscopy (ISIS). A new technique for spatially selective NMR spectroscopy. J Magn Reson 1969;1986(66):283‐294.

[165] Hudsmith LE , Tyler DJ , Emmanuel Y , et al. ^31^P cardiac magnetic resonance spectroscopy during leg exercise at 3 Tesla. Int J Cardiovasc Imaging 2009;25:819‐826. 19697152 10.1007/s10554-009-9492-8

[166] Bakermans AJ , Bazil JN , Nederveen AJ , et al. Human cardiac ^31^P‐MR spectroscopy at 3 Tesla cannot detect failing myocardial energy homeostasis during exercise. Front Physiol 2017;8:939. 29230178 10.3389/fphys.2017.00939 PMC5712006

[167] Yabe T , Mitsunami K , Okada M , Morikawa S , Inubushi T , Kinoshita M . Detection of myocardial ischemia by ^31^P magnetic resonance spectroscopy during handgrip exercise. Circulation 1994;89:1709‐1716. 8149536 10.1161/01.cir.89.4.1709

[168] Buchthal SD , den Hollander JA , Merz CN , et al. Abnormal myocardial phosphorus‐31 nuclear magnetic resonance spectroscopy in women with chest pain but normal coronary angiograms. N Engl J Med 2000;342:829‐835. 10727587 10.1056/NEJM200003233421201

[169] Dass S , Cochlin LE , Suttie JJ , et al. Exacerbation of cardiac energetic impairment during exercise in hypertrophic cardiomyopathy: A potential mechanism for diastolic dysfunction. Eur Heart J 2015;36:1547‐1554. 25990345 10.1093/eurheartj/ehv120

[170] Ferrantini C , Belus A , Piroddi N , Scellini B , Tesi C , Poggesi C . Mechanical and energetic consequences of HCM‐causing mutations. J Cardiovasc Transl Res 2009;2:441‐451. 20560002 10.1007/s12265-009-9131-8

[171] Levelt E , Rodgers CT , Clarke WT , et al. Cardiac energetics, oxygenation, and perfusion during increased workload in patients with type 2 diabetes mellitus. Eur Heart J 2016;37:3461‐3469. 26392437 10.1093/eurheartj/ehv442 PMC5201143

[172] Ellis J , Valkovič L , Purvis LAB , Clarke WT , Rodgers CT . Reproducibility of human cardiac phosphorus MRS (^31^P‐MRS) at 7 T. NMR Biomed 2019;32:e4095. 30924566 10.1002/nbm.4095 PMC6546607

[173] Clarke WT , Hingerl L , Strasser B , Bogner W , Valkovič L , Rodgers CT . Three‐dimensional, 2.5‐minute, 7T phosphorus magnetic resonance spectroscopic imaging of the human heart using concentric rings. NMR Biomed 2023;36:e4813. 35995750 10.1002/nbm.4813 PMC7613900

[174] Tyler A , Ellis J , Lau JYC , et al. Compartment‐based reconstruction of 3D acquisition‐weighted ^31^P cardiac magnetic resonance spectroscopic imaging at 7 T: A reproducibility study. NMR Biomed 2023;36:e4950. 37046414 10.1002/nbm.4950 PMC10658645

[175] Apps A , Valkovič L , Peterzan M , et al. Quantifying the effect of dobutamine stress on myocardial P_i_ and pH in healthy volunteers: A ^31^P MRS study at 7T. Magn Reson Med 2021;85:1147‐1159. 32929770 10.1002/mrm.28494 PMC8239988

[176] Moon RB , Richards JH . Determination of intracellular pH by ^31^P magnetic resonance. J Biol Chem 1973;248:7276‐7278. 4743524

[177] Heijtel DFR , Mutsaerts HJMM , Bakker E , et al. Accuracy and precision of pseudo‐continuous arterial spin labeling perfusion during baseline and hypercapnia: A head‐to‐head comparison with ^15^O H₂O positron emission tomography. Neuroimage 2014;92:182‐192. 24531046 10.1016/j.neuroimage.2014.02.011

[178] Václavů L , Meynart BN , Mutsaerts HJMM , et al. Hemodynamic provocation with acetazolamide shows impaired cerebrovascular reserve in adults with sickle cell disease. Haematologica 2019;104:690‐699. 30523051 10.3324/haematol.2018.206094 PMC6442969

[179] Smith KJ , Ainslie PN . Regulation of cerebral blood flow and metabolism during exercise. Exp Physiol 2017;102:1356‐1371. 28786150 10.1113/EP086249

[180] Tymko MM , Ainslie PN , Smith KJ . Evaluating the methods used for measuring cerebral blood flow at rest and during exercise in humans. Eur J Appl Physiol 2018;118:1527‐1538. 29767351 10.1007/s00421-018-3887-y

[181] Aaslid R , Markwalder TM , Nornes H . Noninvasive transcranial Doppler ultrasound recording of flow velocity in basal cerebral arteries. J Neurosurg 1982;57:769‐774. 7143059 10.3171/jns.1982.57.6.0769

[182] Verbree J , Bronzwaer A , van Buchem MA , Daemen M , van Lieshout JJ , van Osch M . Middle cerebral artery diameter changes during rhythmic handgrip exercise in humans. J Cereb Blood Flow Metab 2017;37:2921‐2927. 27837189 10.1177/0271678X16679419 PMC5536799

[183] Tarumi T , Yamabe T , Fukuie M , et al. Brain blood and cerebrospinal fluid flow dynamics during rhythmic handgrip exercise in young healthy men and women. J Physiol 2021;599:1799‐1813. 33481257 10.1113/JP281063

[184] Alsop DC , Detre JA , Golay X , et al. Recommended implementation of arterial spin‐labeled perfusion MRI for clinical applications: A consensus of the ISMRM perfusion study group and the European consortium for ASL in dementia. Magn Reson Med 2015;73:102‐116. 24715426 10.1002/mrm.25197 PMC4190138

[185] Mast IH , Baas KPA , Jørstad HT , Wood JC , Nederveen AJ , Bakermans AJ . Dynamic MR imaging of cerebral perfusion during bicycling exercise. Neuroimage 2022;250:118961. 35121183 10.1016/j.neuroimage.2022.118961

[186] Hiura M , Nariai T , Ishii K , et al. Changes in cerebral blood flow during steady‐state cycling exercise: A study using oxygen‐15‐labeled water with PET. J Cereb Blood Flow Metab 2014;34:389‐396. 24301294 10.1038/jcbfm.2013.220 PMC3948124

[187] Smith JC , Paulson ES , Cook DB , Verber MD , Tian Q . Detecting changes in human cerebral blood flow after acute exercise using arterial spin labeling: Implications for fMRI. J Neurosci Methods 2010;191:258‐262. 20603148 10.1016/j.jneumeth.2010.06.028

[188] Aghjayan SL , Lesnovskaya A , Esteban‐Cornejo I , Peven JC , Stillman CM , Erickson KI . Aerobic exercise, cardiorespiratory fitness, and the human hippocampus. Hippocampus 2021;31:817‐844. 34101305 10.1002/hipo.23337 PMC8295234

[189] MacIntosh BJ , Crane DE , Sage MD , et al. Impact of a single bout of aerobic exercise on regional brain perfusion and activation responses in healthy young adults. PLoS One 2014;9:e85163. 24416356 10.1371/journal.pone.0085163 PMC3885687

[190] Steventon JJ , Foster C , Furby H , Helme D , Wise RG , Murphy K . Hippocampal blood flow is increased after 20 min of moderate‐intensity exercise. Cereb Cortex 2020;30:525‐533. 31216005 10.1093/cercor/bhz104 PMC7703728

[191] Olivo G , Nilsson J , Garzón B , et al. Immediate effects of a single session of physical exercise on cognition and cerebral blood flow: A randomized controlled study of older adults. Neuroimage 2021;225:117500. 33169699 10.1016/j.neuroimage.2020.117500

[192] Robertson AD , Crane DE , Rajab AS , et al. Exercise intensity modulates the change in cerebral blood flow following aerobic exercise in chronic stroke. Exp Brain Res 2015;233:2467‐2475. 26003127 10.1007/s00221-015-4317-6

[193] Theyers AE , Goldstein BI , Metcalfe AW , Robertson AD , MacIntosh BJ . Cerebrovascular blood oxygenation level dependent pulsatility at baseline and following acute exercise among healthy adolescents. J Cereb Blood Flow Metab 2019;39:1737‐1749. 29561225 10.1177/0271678X18766771 PMC6727139

[194] Toth P , Tarantini S , Csiszar A , Ungvari Z . Functional vascular contributions to cognitive impairment and dementia: Mechanisms and consequences of cerebral autoregulatory dysfunction, endothelial impairment, and neurovascular uncoupling in aging. Am J Physiol Heart Circ Physiol 2017;312:H1‐H20. 27793855 10.1152/ajpheart.00581.2016 PMC5283909

[195] Haeger A , Costa AS , Schulz JB , Reetz K . Cerebral changes improved by physical activity during cognitive decline: A systematic review on MRI studies. Neuroimage Clin 2019;23:101933. 31491837 10.1016/j.nicl.2019.101933 PMC6699421

[196] Billinger SA , Arena R , Bernhardt J , et al. Physical activity and exercise recommendations for stroke survivors: A statement for healthcare professionals from the American Heart Association/American Stroke Association. Stroke 2014;45:2532‐2553. 24846875 10.1161/STR.0000000000000022

[197] Bakermans AJ , Wessel CH , Zheng KH , Groot PFC , Stroes ESG , Nederveen AJ . Dynamic magnetic resonance measurements of calf muscle oxygenation and energy metabolism in peripheral artery disease. J Magn Reson Imaging 2020;51:98‐107. 31218803 10.1002/jmri.26841 PMC6916546

[198] Schwartz M , Steidle G , Martirosian P , et al. Spontaneous mechanical and electrical activities of human calf musculature at rest assessed by repetitive single‐shot diffusion‐weighted MRI and simultaneous surface electromyography. Magn Reson Med 2018;79:2784‐2794. 28921633 10.1002/mrm.26921

[199] Zhang B , Lowrance D , Sarma MK , et al. 3T ^31^P/^1^H calf muscle coil for ^1^H and ^31^P MRI/MRS integrated with NIRS data acquisition. Magn Reson Med 2024;91(6):2638‐2651. 38263948 10.1002/mrm.30025

[200] Morales MA , Assana S , Cai X , et al. An inline deep learning based free‐breathing ECG‐free cine for exercise cardiovascular magnetic resonance. J Cardiovasc Magn Reson 2022;24:47. 35948936 10.1186/s12968-022-00879-9 PMC9367083

[201] Morales MA , Manning WJ , Nezafat R . Present and future innovations in AI and cardiac MRI. Radiology 2024;310:e231269. 38193835 10.1148/radiol.231269 PMC10831479

[202] Schilling M , Unterberg‐Buchwald C , Lotz J , Uecker M . Assessment of deep learning segmentation for real‐time free‐breathing cardiac magnetic resonance imaging at rest and under exercise stress. Sci Rep 2024;14:3754. 38355969 10.1038/s41598-024-54164-z PMC10866998

[203] Tao Y , Lv Z , Liu W , Qi H , Hu P . Recurrent neural network‐based simultaneous cardiac T_1_, T_2_, and T_1ρ_ mapping. NMR Biomed 2024;e5133. 10.1002/nbm.5133. 38520183

[204] Halfmann MC , Müller L , von Henning U , et al. Cardiac MRI‐based right‐to‐left ventricular blood pool T_2_ relaxation times ratio correlates with exercise capacity in patients with chronic heart failure. J Cardiovasc Magn Reson 2023;25:33. 37331991 10.1186/s12968-023-00943-y PMC10278263

[205] Campbell‐Washburn AE , Varghese J , Nayak KS , Ramasawmy R , Simonetti OP . Cardiac MRI at low field strengths. J Magn Reson Imaging 2024;59:412‐430. 37530545 10.1002/jmri.28890 PMC10834858

[206] Seemann F , Javed A , Khan JM , et al. Dynamic lung water MRI during exercise stress. Magn Reson Med 2023;90:1396‐1413. 37288601 10.1002/mrm.29716 PMC10521349

[207] Burrage MK , Hundertmark M , Valkovič L , et al. Energetic basis for exercise‐induced pulmonary congestion in heart failure with preserved ejection fraction. Circulation 2021;144:1664‐1678. 34743560 10.1161/CIRCULATIONAHA.121.054858 PMC8601674

[208] Jezek F , Randall EB , Carlson BE , Beard DA . Systems analysis of the mechanisms governing the cardiovascular response to changes in posture and in peripheral demand during exercise. J Mol Cell Cardiol 2022;163:33‐55. 34626617 10.1016/j.yjmcc.2021.09.013

[209] Tian Q , Bilgel M , Walker KA , et al. Skeletal muscle mitochondrial function predicts cognitive impairment and is associated with biomarkers of Alzheimer's disease and neurodegeneration. Alzheimers Dement 2023;19:4436‐4445. 37530130 10.1002/alz.13388 PMC10592411

[210] Jeneson JAL , Bruggeman FJ . Robust homeostatic control of quadriceps pH during natural locomotor activity in man. FASEB J 2004;18:1010‐1012. 15059964 10.1096/fj.03-0762fje

[211] Ogoh S . Cardiac output‐mediated regulation of cerebral blood flow during exercise: Clinical perspectives on the indirect impact of muscle metaboreflex. Exp Physiol 2024. 10.1113/EP091591. PMC12053890 38500291

